# Distilling First-Principles Accuracy into Compact Machine Learning Potentials for Condensed-Phase Chemistry

**DOI:** 10.1021/acs.jctc.6c01131

**Published:** 2026-10-09

**Authors:** Sijia Chen, Niamh O’Neill, Benjamin X. Shi, Venkat Kapil

**Affiliations:** † 525571 Initiative for Computational Catalysis, Flatiron Institute, New York, New York 10010, United States; ‡ 2152 Max Planck Institute for Polymer Research, Mainz 55128, Germany; § Department of Physics and Astronomy, University College London, 7-19 Gordon St, London WC1H 0AH, U.K.; ∥ 27218 Thomas Young Centre and London Centre for Nanotechnology, 9 Gordon St, London WC1H 0AH, U.K.

## Abstract

Accurate machine learning interatomic potentials (MLIPs) have made first-principles-quality potential energy surfaces increasingly accessible for condensed-phase chemistry, but their inference cost can still limit the sampling needed to compute experimentally relevant observables. In this work, we use knowledge distillation to construct compact “student” models that retain the accuracy of much larger “teacher” models obtained through either finetuning of foundation models or Δ-learning. The resulting students reduce production simulation cost by approximately an order of magnitude, making high-accuracy sampling practical for challenging condensed-phase problems. We demonstrate this across three problems of increasing sampling complexity: finite-temperature *NPT* simulations of ice Ih, classical and path-integral simulations of liquid water over 240–370 K, and path-integral umbrella-sampling simulations of water dissociation at the anatase TiO_2_(101)/water interface. When compared, the distilled students reproduce the target observables of their teachers more reliably than equally compact models trained only on the available reference data. The liquid water student, distilled from a Δ-learned CCSD­(T)-quality teacher, reproduces thermodynamic, structural, transport, and nuclear quantum effects over the full temperature range studied. At the TiO_2_/water interface, knowledge distillation makes PIMD umbrella sampling practical and shows that nuclear quantum effects lower the dissociation barrier by approximately 2 kcal/mol and shift the molecular–dissociated free energy difference into closer agreement with recent solid-state ^17^O NMR measurements. Our work demonstrates how knowledge distillation can make accurate MLIPs practical for the sampling methods needed to connect condensed-phase reaction thermodynamics with experiment, notably for interfacial chemistry and catalysis.

## Graphical abstract

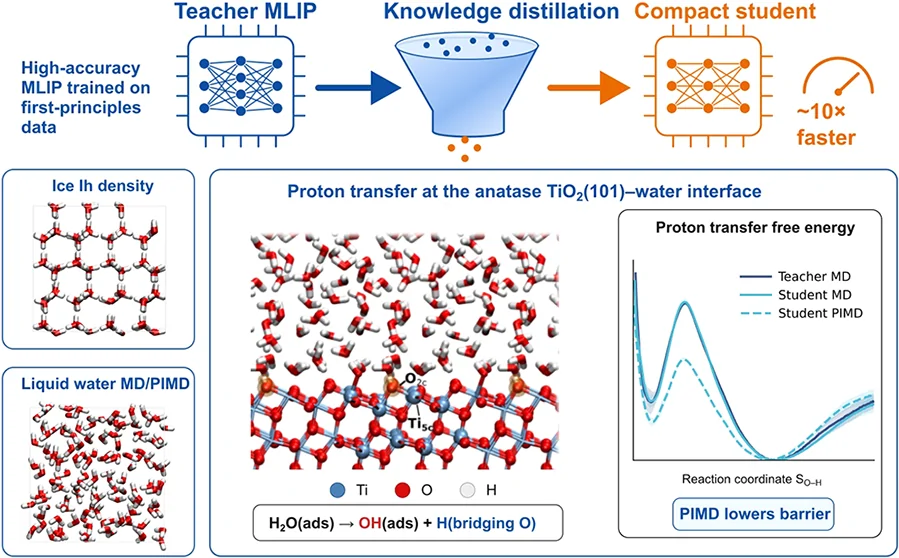

## 1 Introduction

Machine learning interatomic potentials (MLIPs)^1,2^ have become a cornerstone of chemical and materials science. However, despite greatly reducing the computational cost of sampling first-principles quality potential energy surfaces, many problems of interest are still computationally demanding. This limitation arises because the model must simultaneously satisfy two stringent requirements. The machine learning architecture must be expressive enough to accurately describe a wide region of configuration space relevant to a problem but also efficient enough to sample this space in a feasible amount of computational time.^3–6^ Due to the general inverse relation between expressivity and efficiency, a number of challenging regimes relevant to chemical and materials simulations arise, including long molecular dynamics (MD) trajectories,^7^ enhanced sampling of rare events such as bond breaking,^8^ and path-integral (PI) MD simulations that account for nuclear quantum effects (NQEs).^9^

In the past few years, there has been significant progress in improving the accuracy of MLIPs across configurational and alchemical space.^10^ Besides the development of new graph neural network architectures,^11,12^ transfer learning^13^—adapting or exploiting the weights of a pretrained model for data associated with a new problem—has proven advantageous over training a new model from scratch on the same data. This approach has enabled MLIPs with beyond-DFT accuracy developed by finetuning a model trained on DFT to small volumes of beyond-DFT data^14,15^ or by augmenting a model trained on DFT with a differential learning^16^ model trained on the difference between DFT and beyond-DFT.^17–20^ More recently, transfer learning has simplified the development of accurate MLIPs at (beyond-)­DFT accuracy for generic systems by applying various types of finetuning,^21–25^ or in some cases, differential learning,^26,27^ to large models trained on diverse datasets, referred to as foundation MLIPs.^28–34^ While foundation MLIPs expedite model development by reducing human effort and volume of training data, their inference cost is typically much higher than that of a carefully curated small MLIP with a simpler architecture trained on a large amount of data.^35–40^ As a result, their application to large systems (thousands to millions of atoms), long simulations (nanoseconds to milliseconds^7^), or sophisticated sampling algorithms remains challenging.

More recently, efforts have intensified toward reducing the computational cost of MLIPs, ideally at constant accuracy. An attractive approach, among others, is knowledge distillation, a strategy widely used in computer science.^41–43^ When applied to MLIPs, a large and/or broadly applicable so-called “teacher” model is used to generate training data relevant to the problem of interest at the cost of MLIP inference rather than additional first-principles calculations, and a compact “student” model with a low inference cost is then trained to fit the problem-specific data. Teacher information can also be incorporated through auxiliary targets beyond the total energies and forces conventionally used to train MLIPs. For example, recent studies have trained compact students to match the energy Hessians of teacher foundation models,^44^ teacher-derived atomwise energy decompositions,^45^ or ensemble-averaged teacher forces for energy-only high-level datasets.^46^ In the context of condensed-phase simulations, knowledge distillation has been successfully demonstrated to reduce the inference cost of foundation MLIPs for specific classes of systems^47,48^ and has also been explored to accelerate molecular dynamics driven by finetuned foundation MLIPs for materials.^49,50^

Beyond the choice of training targets, applying knowledge distillation to problems that require demanding production sampling remains nontrivial because it is difficult to generate an exhaustive dataset *a priori*. The student model must then be accurate not only on the distillation set but also for out-of-distribution configurations encountered during production simulations, while still retaining low inference cost to maintain the computational advantage of knowledge distillation. Some examples include chemical reactions in the bulk or at interfaces where a failure of knowledge distillation has been noted.^50^ In this case, knowledge distillation would in principle require thorough sampling of transition-state regions in the training set, but generating such data with the teacher model can be computationally prohibitive. Another is the incorporation of NQEs, which requires expensive PIMD sampling of regions of configuration space (due to zero-point motion and tunneling) that are much less accessible or not accessible to classical simulations. In such cases, the challenge is not only how to distill a compact and efficient student but also how to obtain an accurate teacher without requiring a large first-principles training set, since the accuracy ultimately achievable by the student is inherited from the teacher. Thus, an understanding of how accurate the teacher must first be, how much of the relevant configuration space the student must see during training, and how expressive the student model itself must be would be valuable for enabling fast and accurate MLIPs for challenging condensed-phase chemistry.

In this work, we combine transfer learning and knowledge distillation to show that compact MLIPs can be made accurate and computationally efficient, while remaining applicable beyond the regimes explicitly represented in the training set. We demonstrate this on three condensed-phase problems of progressively increasing difficulty: (1) ice Ih, where a finetuned teacher is distilled into a student that is nearly 10 times faster while accurately predicting the *NPT* density at thermodynamic state points outside the training regime; (2) liquid water, where a student distilled from a larger Δ-learned CCSD­(T)-accurate teacher is fast enough to explore the temperature dependence of the density isobar, diffusion coefficient and quantum kinetic energies over 240–370 K while incorporating quantum nuclear effects (each requiring large-scale, computationally intensive simulations to converge accurately) in excellent agreement with the experiments; and (3) proton transfer at the anatase TiO_2_(101)/water interface, where a student distilled from a DFT finetuned teacher is about 10 times faster and enables us to examine how quantum nuclear motion modifies the reaction free energy landscape in qualitative agreement with an experiment. In each case, we analyse, where relevant, how the teacher should be constructed through transfer learning from limited reference data, how the distillation training set should be sampled, and how the student model should be chosen to provide the most useful balance between expressivity and simulation throughput for a problem at hand. We find that these trade-offs become progressively tighter as the target problem moves from predicting bulk thermodynamic properties to problems requiring enhanced sampling and/or quantum nuclear motion, but that they nevertheless remain navigable. Overall, our results show that knowledge distillation can make many condensed-phase chemistry problems tractable that would otherwise be limited by the cost of finetuned or Δ-learned models.

## 2 Results and Discussion

We consider three condensed-phase systems of progressively increasing difficulty: a crystalline solid in the *NPT* ensemble, a molecular liquid with quantum nuclear effects, and a reactive solid–liquid interface with quantum nuclear effects. While we briefly summarize the methodology and focus on the key results below, we refer the reader to Section 4 for the associated computational details.

### 2.1 Predicting the Density of Ice Ih

We first demonstrate the combination of transfer learning and knowledge distillation on a simple solid but for a challenging observable, within and outside the regime represented in the training set. We consider the ambient-pressure density of ice Ih, a prototypical system that has served as a test bed for general molecular systems.^21^ Methods capable of resolving the finite-temperature properties of ice Ih in the constant-pressure ensemble are applicable to a much broader class of organic molecular crystals.^51^ Moreover, density is a particularly challenging observable to converge with MLIPs, and small energy and force errors alone do not guarantee its accurate prediction.^21,52^

We consider the dataset of Kaur et al.,^21^ which contains 500 configurations sampled randomly from first-principles molecular dynamics simulations at 100 K and 1 bar using coarsely converged DFT settings. We first obtain an accurate teacher model by finetuning the MACE-MP-0a­(L) foundation model on 400 configurations from this dataset labeled with revPBE-D3(0) total energies and forces, achieving an energy RMSE below 0.01 meV/atom and a force RMSE of 1.04 meV/Å on the remaining 100 configurations used as a test set. Figures S1–S4 show the parity and violin plots of the energy and force predictions and errors on the test set and demonstrate high fidelity across the full dataset. We test this model on the density at 100 K and 1 bar by performing NPT simulations. We obtain a density of 0.9223 ± 0.0002 g cm^–3^, in quantitative agreement with the MLIP and DFT reference densities reported in Kaur et al.,^21^ thereby demonstrating the capability of transfer learning in developing an accurate model. However, the finetuned MACE-MP-0a­(L) architecture is relatively large and computationally demanding (with 780,698 parameters) owing to its use of *L* = 2 spherical harmonics and 128 channels, and achieves only 0.72 ns/day of MD throughput on our 384-atom ice Ih supercell with one A100 GPU (Figure 1b). Since a model of this capacity is intended to describe systems across the full periodic table, it is natural to distill it into a more compact model specialized to ice Ih at ambient pressure.

**Figure 1:**
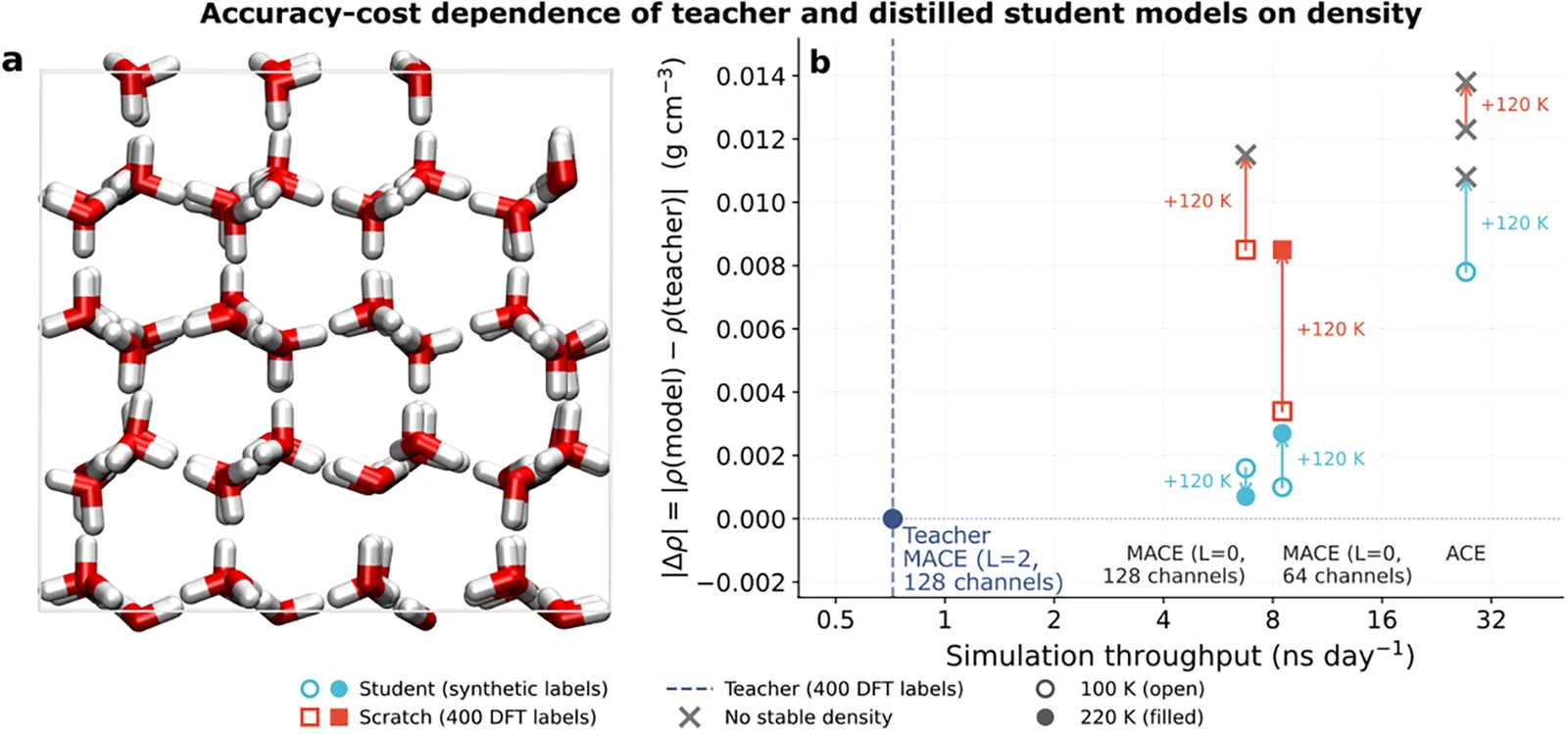
Speed–accuracy trade-off for ice Ih. (a) Representative atomistic configuration of the 384-atom ice Ih supercell used for the NPT simulations, with oxygen atoms in red and hydrogen atoms in white. (b) Absolute deviation of the predicted density from the finetuned teacher, |Δρ| = |ρ_model_ – ρ_teacher_|, versus simulation throughput (ns day^–1^, log_2_ scale). The teacher (dark blue dashed reference line and dot; labeled “MACE, *L* = 2, 128 channels”) is the finetuned MACE-MP-0a­(L) foundation model and defines |Δρ| = 0 at its measured throughput. The three small student architectures (labeled ”MACE, *L* = 0, 128 channels”, “MACE, *L* = 0, 64 channels”, and “ACE”) are trained on either 2000 teacher-labeled configurations (Student, cyan circles) or the original 400 DFT configurations (Scratch, orange-red squares). Open markers correspond to 100 K, 1 bar; filled markers to 220 K, 1 bar. Vertical segments connect the two temperatures of the same model. Configurations that fail to sustain stable NPT dynamics with the default 0.5 fs timestep are marked as “No stable density” (gray X) and stacked above the architecture’s stable points. All simulations use a 384-atom supercell on a single NVIDIA A100-PCIe GPU; replica-to-replica standard deviations of the density are below 0.0003 g cm^–3^ for all stable runs and are smaller than the symbol size.

To generate the training set for knowledge distillation, we consider the original dataset with 400 configurations and add 1600 new configurations by rattling the former, as was proposed by Gardner et al.^50^ The reasoning behind rattling (instead of performing a simulation with the teacher model) is that ice Ih is solid and its thermal fluctuations at low temperatures would mimic a Gaussian distribution. Because training and validation are comparatively inexpensive for ice Ih, we explore three student architectures spanning a broad accuracy-cost range: (1) a MACE model with invariant (*L* = 0) messages and 128 channels, comprising 460,432 parameters (this model has the same number of channels as the teacher but uses invariant messages), (2) a MACE model with invariant (*L* = 0) messages and 64 channels, comprising 148,944 parameters (this model has half as many channels as the teacher and invariant messages), (3) an ACE model with a Finnis–Sinclair two-density embedding, up to 1000 basis functions per element, and body orders up to 6, comprising 5,296 parameters (a simpler and relatively inexpensive architecture). Energy and force errors on a held-out test set for all student and scratch architectures are reported in Figures S1–S4.

In Figure 1, we report the error in the density at 100 K and 1 bar relative to the teacher model for the three student architectures. As a point of comparison, we also include the corresponding results for models of the same architectures trained from scratch on the original 400-configuration dataset, together with their computational cost. For the least expressive but most computationally efficient ACE models, the model trained from scratch is unstable in production MD, whereas the distilled student, although stabilized by training on a larger volume of data, yields the largest density error among the student models. By contrast, the more expressive MACE student models, although approximately four times slower than the ACE student, recover the density to within 0.002 g cm^–3^. The corresponding MACE models trained from scratch, however, exhibit larger errors, with the more expressive 128-channel model again performing worse. Although these results already show that, at 100 K, the student models outperform equally sized models trained from scratch, this analysis does not fully reveal the benefits of finetuning and distillation. We therefore also evaluate the density error at 220 K, in a region of configuration space not seen during student training, relative to the teacher. Under the standard 0.5 fs timestep, both ACE models, whether distilled or trained from scratch, are numerically unstable. Among the MACE models trained from scratch, the 64-channel model exhibits an approximately twofold increase in the density error at 220 K, whereas the 128-channel model becomes unstable. A plausible explanation is that the 128-channel scratch model’s larger parameter count is underdetermined by only 400 training configurations, leaving rough PES features off-distribution that destabilize NPT dynamics at 220 K. By contrast, the distilled student models retain errors of the same order of magnitude as at 100 K, indicating robust generalizability beyond the regime represented in the training set.

Separately, to assess whether direct finetuning of a smaller foundation model can provide an accurate teacher, we finetuned the 128-channel *L* = 0 MACE-MP-0a­(S) foundation model on nested subsets containing 50, 100, 200, and 400 of the same DFT configurations, denoted S-50, S-100, S-200, and S-400. For comparison, L-50 and L-400 denote the 128-channel *L* = 2 MACE-MP-0a­(L) foundation model finetuned on 50 and 400 configurations, respectively. As shown in Figure S5 and Table S1, the density shifts from underprediction with S-50, through close agreement with S-100 and S-200, to a small overprediction with S-400. By contrast, L-50 reproduces the L-400 density at both temperatures, consistent with the data-efficient finetuning of the large foundation model reported by Kaur et al.^21^ The large foundation model therefore reaches the target density regime with fewer DFT-labeled configurations under the tested protocol. Furthermore, it can be distilled to a 64-channel *L* = 0 model that is faster than the S series models.

These results yielded three lessons. First, distillation can produce compact models that are both efficient and accurate while retaining sufficient generalizability to remain reliable slightly outside the regime represented in the training set. In the case of solids, this may be achieved by expanding the reference training set efficiently through rattling and relabeling. Second, the most useful student is not necessarily the cheapest or least expressive. Although a very inexpensive model may be sufficient when no exploration beyond the training set is required, in the present case, the 64-channel MACE student provides the best balance among accuracy, generalizability, and cost, reproducing the teacher’s density predictions at both 100 and 220 K while remaining approximately 10 times faster than the teacher. Third, applying transfer learning to a large foundation model, followed by distillation to a smaller model, constitutes a more training-data-efficient approach and provides better accuracy-cost trade-off than applying transfer learning directly to models with smaller capacity.

### 2.2 Quantum Nuclear Effects in Ambient Pressure Water

We next simulate static and dynamical properties of liquid water at 1 bar while explicitly incorporating quantum nuclear effects. This is a more challenging case for the combined transfer learning and distillation strategy for two reasons. First, relative to a solid, the liquid spans a broader, more flexible, and more temperature-dependent configuration space, which is further expanded by quantum delocalization of protons.^64^ This requires a distilled student that is both sufficiently expressive and efficient. Second, the properties of liquid water are highly sensitive to the underlying potential energy surface, including the choice of DFT functional and dispersion correction.^65^ This in turn requires a correspondingly accurate teacher. To address these challenges, we use the CCSD­(T)-accurate teacher model of O’Neill et al.,^19^ in which a short-ranged differential learning MACE model trained on CCSD­(T)*-*DFT energy differences augments a DFT-trained baseline MACE model. The original work explored the classical structural properties of water over the range 250 to 330 K. Classical transport properties and the impact of quantum nuclear effects on structural and dynamical properties were considered only at 298 K due to the high combined cost of the baseline and learned differential potentials and the 64 times higher computational cost of PIMD than standard MD. We therefore distill this composite teacher, which requires evaluation of both the baseline (128-channel, *L* = 0) and Δ MACE models (64-channel, *L* = 0), into a single student. Instead of using a MACE architecture for the student, we consider an ACE model with a substantially lower inference cost for long classical and path-integral simulations across the temperature range studied compared to the teacher.

We construct the distillation dataset from equilibrium NVT simulations with the teacher model at 260, 300, 330, and 360 K and densities of 0.85, 0.90, 0.95, 1.00, 1.05, 1.10, and 1.15 g/cm^3^, and from equilibrium NPT simulations at the same temperatures and pressures of −1500, −500, −300, 1, 2, 500, 1000, 4000, and 8000 bar. Each simulation is run for 0.5 ns, provided that it remains stable. To improve coverage of configurations relevant to NQEs, we additionally include 50 ps PIMD NVT simulations at 300 K over densities of 0.85, 0.90, 0.95, 1.00, 1.05, and 1.10 g/cm^3^. From these trajectories, we select 10,000 teacher-labeled configurations to train a student ACE model using a random 9:1 training:validation split. This ACE model is parametrized with a Finnis–Sinclair six-density embedding and up to 1000 basis functions per element, and yields a simulation throughput speed-up of 10 compared to the teacher model. On the validation set, the student model achieves an energy MAE and RMSE of 0.79 and 0.82 meV/atom, respectively, and a force MAE and RMSE of 13.35 and 19.04 meV/Å, respectively, with respect to the teacher model. The validation-set errors are nearly identical to the training-set errors, indicating faithful reproduction of the teacher without signs of overfitting. As a primary validation, we compare the O–O and O–H radial distribution functions at 300 K obtained with MD and PIMD for the student and teacher models, as shown in Figure S6 of the SI. We note that the student reproduces the teacher RDFs closely for both classical and quantum nuclei. The agreement is especially close for the quantum results, where the student and teacher curves are nearly indistinguishable. For the classical RDFs, the student also closely follows the teacher, with the most noticeable difference being a slightly lower first peak in the student O–O RDF.

Having established confidence in the structure of water at ambient conditions, we explore the density of water in the 240–370 K range at 1 bar. To this end, we perform MD and PIMD simulations in the *NPT* ensemble. At each temperature, five independent MD replicas are run until the density standard deviation falls below 0.001 g/cm^3^, requiring trajectory lengths between 2 ns at high temperature and 20 ns at 240 K. For PIMD, we perform five independent *NPT* simulations at each temperature using 32 beads, with trajectory lengths of up to 2 ns per replica. In Figure 2, we compare the MD and PIMD properties of liquid water predicted by the distilled student with the experiment and the density predicted by the teacher model, taken from ref 19, which includes MD simulations from 250 to 370 K and only one PIMD simulation at 298 K. For the density isobar (Figure 2b), the student closely reproduces the teacher references at 298 K to within statistical error. We also note that the student reproduces the teacher within statistical uncertainty at temperatures above 280 K, but overestimates the density as the temperature decreases, by up to 0.025 g/cm^3^ at 250 K.

**Figure 2:**
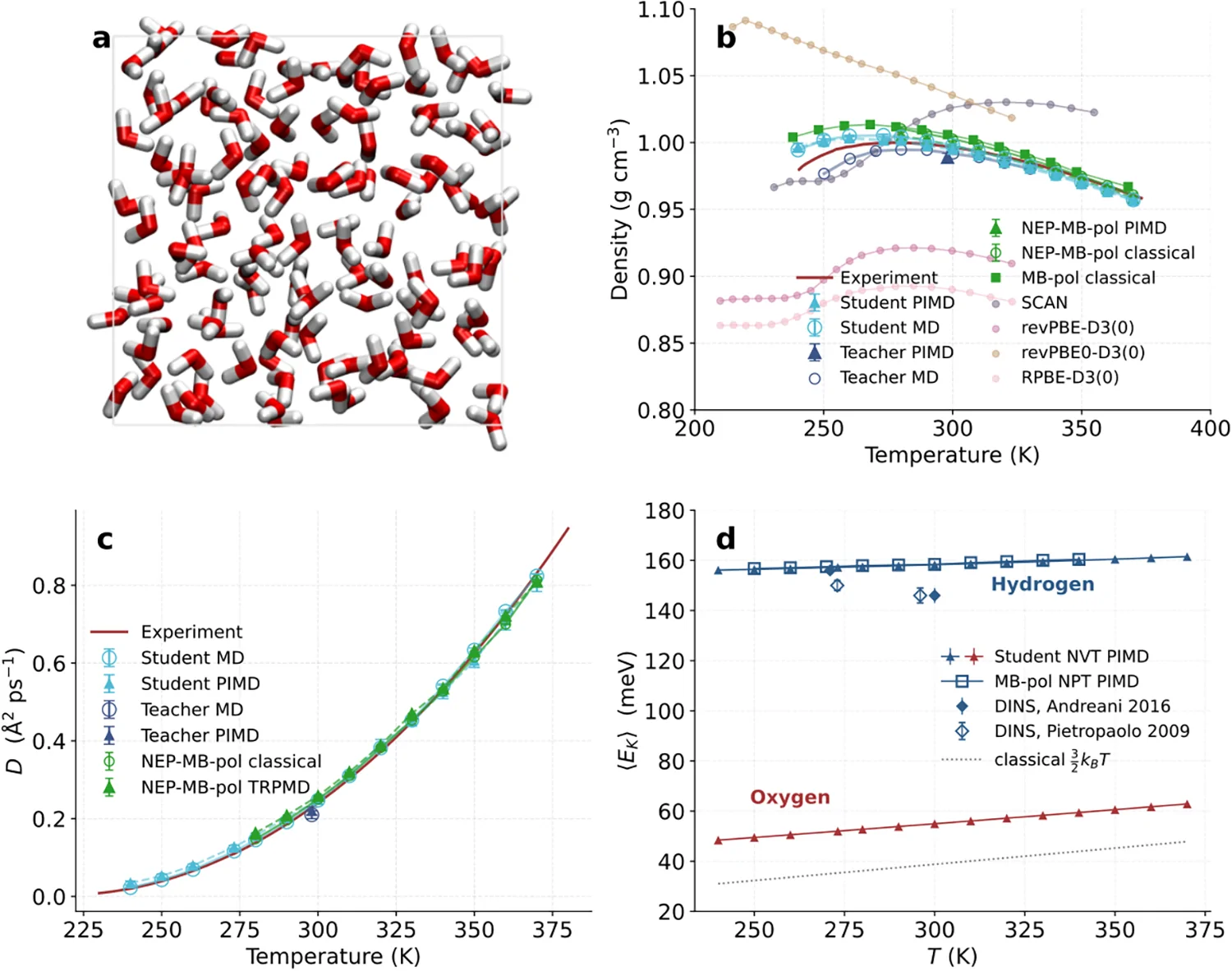
Thermodynamic and dynamical properties of liquid water, with and without quantum nuclear effects, predicted by the student model, compared with experiment and other simulations. (a) Representative atomistic snapshot of a liquid water configuration from the production simulations, with oxygen atoms in red and hydrogen atoms in white. (b) Density isobar. Classical molecular dynamics (MD) and path-integral MD (PIMD) results for the student and teacher are compared with several classical-nuclei DFT references^53^ and experiment.^54–56^ Teacher data are reproduced from O’Neill et al.^19^ All D3(0) curves correspond to the zero-damping variant of the Grimme dispersion correction.^57^ (c) Self-diffusion coefficient as a function of temperature, corrected for finite-size effects.^58^ Experimental data are from refs 59,60 . (d) Mean nuclear quantum kinetic energy ⟨*E*_*K*_⟩ of hydrogen and oxygen atoms from NVT PIMD simulations (64 beads), compared with MB-pol NPT PIMD results from Kapil et al.^61^ and deep inelastic neutron scattering (DINS) measurements.^62,63^ The classical equipartition limit $\frac{3}{2}k_{B}T$ is shown as a dashed line for each species. Classical MD results are plotted with circles, while path-integral results are plotted with triangles (PIMD for equilibrium properties and TRPMD for self-diffusion). Lines between symbols are a guide to the eye.

To examine whether training-data composition contributes to this discrepancy, we retrain the same ACE architecture at a fixed total budget of 10,000 teacher-labeled configurations, replacing 2000 configurations drawn at 300, 330, and 360 K with 2000 configurations drawn at 240, 250, and 280 K. This low-temperature enrichment improves student–teacher density agreement in the low-temperature region (Figure S7 and Table S4), showing that optimization of the training-data composition can improve the student’s reproduction of a teacher-level observable. However, because the student capacity is held fixed and the training-data composition is not exhaustively optimized, the remaining difference may reflect incomplete configurational coverage, limited student capacity, or both. Nonetheless, the student does not deviate from the experiment more than the teacher does and also exhibits an improvement over the predictions of MLIPs trained on DFT.

The reduced computational cost of the student further enables extension of PIMD simulations across the full 240–370 K range. As shown in Figure 2, the inclusion of NQEs leads to a small reduction in density, on the order of ∼0.004 g/cm^3^ near room temperature and of similar magnitude across the temperature range studied. Numerical density values from the classical NPT and PIMD NPT isobars are tabulated in Supporting Information (SI) Tables S2 and S3. This subtle difference is noteworthy, as it is consistent with the experimentally observed difference between the densities of H_2_O and D_2_O after rescaling D_2_O by the mass ratio *M*_H_2_O_/*M*_D_2_O_ (SI Table S5). Across the 240–370 K range studied, the student’s density accuracy is comparable to that of MB-pol^66^ and its neuroevolution surrogate NEP-MB-pol,^67^ two of the most accurate molecular models of water currently available. We next study the self-diffusion coefficient of water with finite-size corrections, which was only simulated at 298 K with the teacher model by O’Neill et al.^19^ Exploiting the computational efficiency of the student model, we are able to estimate the self-diffusion coefficient with MD and TRPMD across the full 240–370 K range (Figure 2c). At 300 K, the predicted diffusion coefficient agrees with both the teacher and experiment. Across 240–370 K, the student remains in close agreement with the experimental temperature dependence, with deviations below 5%. Although experimental self-diffusion coefficients are available for D_2_O, rescaling them to remove the trivial mass dependence and obtain an H_2_O-equivalent reference is nontrivial. We therefore do not make a direct comparison with experimental D_2_O diffusion data. Numerical diffusion coefficients from the classical NVT and TRPMD NVT trajectories are tabulated in SI Tables S6 and S7.

Across the temperature range studied, the classical and TRPMD diffusion coefficients agree within statistical uncertainty, indicating that NQEs have a small effect on water’s self-diffusion. This is consistent with NEP-MB-pol classical and TRPMD simulations,^67^ which similarly show only a small change in the self-diffusion coefficient upon inclusion of NQEs. By contrast, *ab initio* path-integral simulations of liquid water have reported NQE-induced reductions of the self-diffusion coefficient of approximately 30% for the revPBE-D3 GGA functional and 14% for the revPBE0-D3 hybrid functional,^68^ illustrating that the apparent magnitude of NQEs on water dynamics depends sensitively on the underlying potential energy surface.

We also consider an observable not previously reported for the teacher model and directly sensitive to quantum nuclear motion: the mean nuclear kinetic energy of O and H atoms, ⟨*E*_*K*_⟩ (Figure 2d). In contrast to density and diffusion, for which NQEs produce relatively modest changes, ⟨*E*_*K*_⟩ differs qualitatively from its classical counterpart. For hydrogen, the student predicts ⟨*E*_*K*_⟩ = 157.2 meV at 273 K and 158.3 meV at 300 K, exceeding the classical equipartition value by more than a factor of four due to zero-point motion in the high-frequency intramolecular and librational modes. The corresponding oxygen kinetic energy is much closer to the classical limit, consistent with its higher mass. These values are in quantitative agreement with MB-pol PIMD benchmarks^61,69^ and with deep inelastic neutron scattering (DINS) measurements^62^ in the supercooled regime, which report ⟨*E*_*K*_⟩H = 156 ± 2 meV at 271 K. At room temperature, however, DINS experiments yield a lower value of 146±3 meV, highlighting a known discrepancy between experiment and PIMD simulations that remains unresolved.^61^ The close agreement with both MB-pol and low-temperature experimental data indicates that the student accurately captures the quantum nuclear fluctuations of O–H bonds in water. Bead-convergence tests and the temperature-resolved ⟨*E*_*K*_⟩ values across 240–370 K are reported in SI Tables S8 and S9.

This system shows that distillation can transfer the accuracy of an expensive composite teacher (here, a baseline + Δ MACE) into a single compact student (ACE, ∼10 × faster) without loss of thermodynamic, structural, transport, or quantum nuclear accuracy across a temperature range substantially broader than the range that can routinely be accessed with the teacher model.

### 2.3 Proton Transfer at the TiO_2_-Water Interface

Finally, we test the combined finetuning and knowledge distillation protocol to study the role of quantum nuclear motion on water dissociation at the TiO_2_ interface. This problem provides a stringent test case because it requires accurately capturing bond breaking and formation in the presence of quantum proton fluctuations at a chemically diverse interface. The TiO_2_-water interface is an intensively studied model system for photocatalysis,^71–73^ photoelectrochemistry,^71,72^ environmental remediation,^73^ wetting,^74,75^ and biomaterials.^76,77^ Over the past few decades, experimental work has progressed from ultrahigh-vacuum studies of well-defined single-crystal surfaces to increasingly realistic investigations of hydrated and fully aqueous interfaces using ambient-pressure and surface-sensitive probes.^78–80^ These studies have shown that the interfacial speciation of water on TiO_2_ is sensitive to surface orientation, defects, hydroxylation, coverage, and the surrounding hydrogen-bond network.^78,79^ State-of-the-art simulations of water dissociation at the TiO_2_ interface have been performed by Zeng et al.^70^ utilizing MLIPs with enhanced sampling to estimate the classical free energy surface associated with water dissociation. Given the important role of quantum nuclear effects in proton-transfer reactions,^64^ and the nontrivial influence of interfaces on the stabilization of protonated and hydroxylated species, we study how quantum nuclear motion modulates the thermodynamics of water dissociation at the TiO_2_ interface. Dissociation is investigated using the same collective variable *S*_O–H_ adopted in earlier work on this system,^70,81^ a soft-min approximation to the minimum distance between a particular surface oxygen atom and any hydrogen atom in the system (full definition in Section 4). Values of *S*_O–H_ ≈ 1 Å correspond to the dissociated state, where a proton has bonded directly to this surface oxygen, and *S*_O–H_ ≈ 1.67 Å corresponds to the molecularly adsorbed state.

Due to the high cost of enhanced-sampling simulations, the substantial overhead of path-integral simulations, and the sensitivity of proton delocalization to the accuracy of the potential energy surface,^82^ an approach that combines accuracy with computational efficiency is necessary. To this end, we first obtain an accurate teacher model. Rather than using the MLIP of Zeng et al.^70^ directly as the teacher, which was trained on over 10,000 configurations, we instead exploit the data efficiency and robustness of transfer learning. We finetune the 128-channel, *L* = 2 MACE-MP-0a­(L) foundation model on subsets of that dataset and reproduce the classical free energy surface of water dissociation. Element-resolved force parity plots for the finetuned models are reported in Figures S8–S11. In Figure S12 of the SI, we also compare the density and orientation distributions with the reference produced by Zeng et al.^70^ and find that all finetuned models are in agreement. In Figure 3, we further estimate the classical free energy profiles for water dissociation obtained with the different training subsets using umbrella sampling simulations with 18 umbrella centers and 1 ns long MD per window (more details in Section 4). We observe only a weak dependence of the free energies of the reactant and barrier relative to the product on training-set size, with the model trained on 500 configurations reproducing the results of the model finetuned on the full dataset to within a fraction of a kcal/mol.

**Figure 3:**
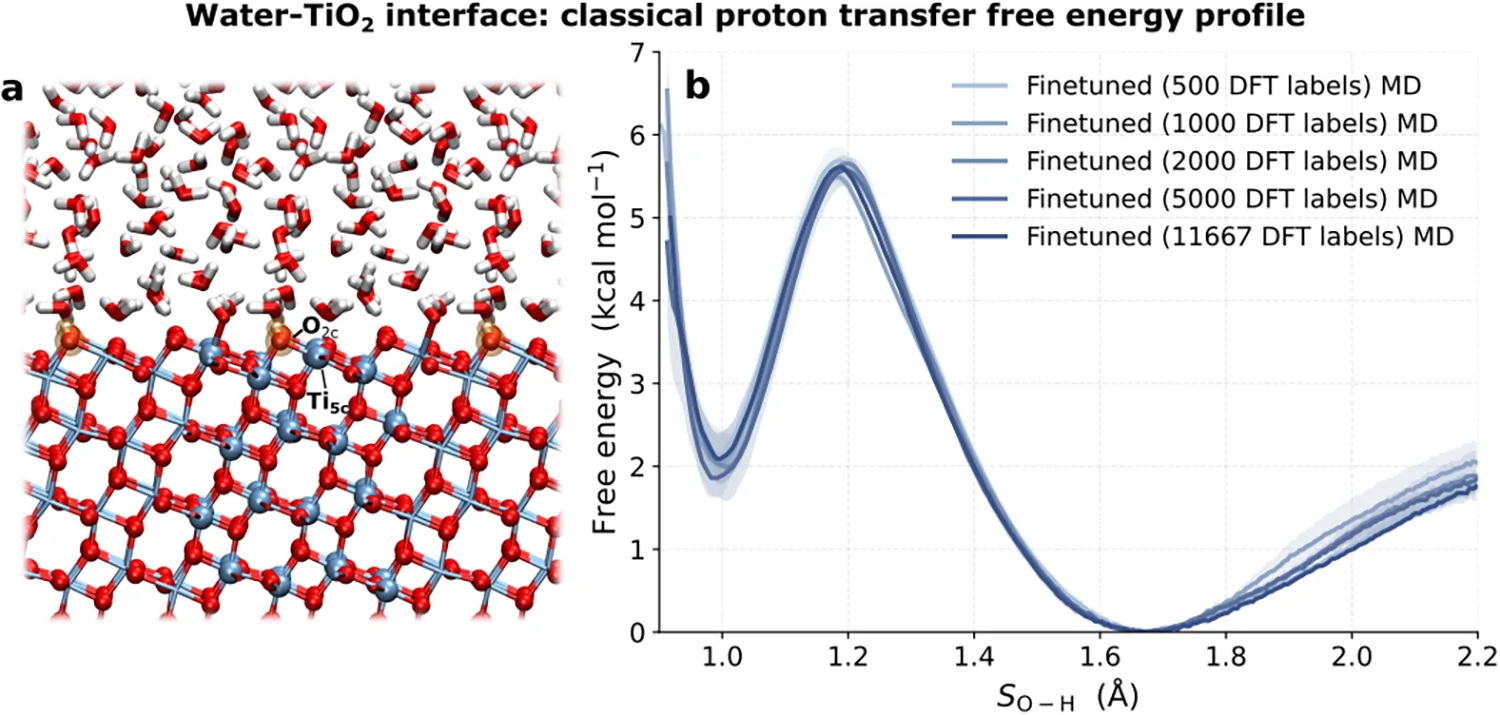
(a) Representative atomistic snapshot of the anatase TiO_2_(101)–water interface used for the umbrella sampling simulations, showing the slab with the adsorbed water layer above. Titanium atoms are shown in blue, oxygen atoms in red, and hydrogen atoms in white. (b) Free energy profiles for water dissociation on the pristine anatase TiO_2_(101)–water interface from umbrella sampling, using large MACE foundation models finetuned on different numbers of DFT configurations drawn from the Zeng et al. dataset.^70^ The minimum near *S*_O–H_ ≈ 1.67 Å corresponds to the molecularly adsorbed state, and the minimum near *S*_O–H_ ≈ 1 Å corresponds to the dissociated state. Curves are colored from light to dark blue with increasing training-set size. Shaded regions denote statistical uncertainty estimated by block averaging.

The corresponding free energy differences and barriers are summarized in Table 1.

**Table 1:** Free Energy Difference between the Molecularly Adsorbed and Dissociated States, Δ*G*, and the Free Energy Barrier, *G* ^‡^, for Water Dissociation on the Anatase TiO_2_(101)–Water Interface Predicted by Different Models

Model	Δ*G* (kcal/mol)	*G* ^‡^ (kcal/mol)
Zeng et al.	1.8 ± 0.3	5.4 ± 0.4
Finetuned (500 DFT labels)	2.1 ± 0.3	5.7 ± 0.2
Finetuned (1000 DFT labels)	2.0 ± 0.4	5.5 ± 0.1
Finetuned (2000 DFT labels)	2.0 ± 0.1	5.7 ± 0.1
Finetuned (5000 DFT labels)	1.9 ± 0.2	5.6 ± 0.2
Finetuned (11,667 DFT labels)	2.1 ± 0.3	5.6 ± 0.1
Classical student	2.1 ± 0.2	5.5 ± 0.1
Small MACE (1000 DFT)	0.9 ± 0.4	4.6 ± 0.1
Quantum student	1.3 ± 0.1	3.5 ± 0.1

Having established a data-efficient route to obtain a teacher model, we next ask whether the classical free energy landscape of the MACE-MP-0a­(L) model finetuned on 1000 DFT configurations can be transferred to a compact student. We directly consider a MACE model with *L* = 0 and 64 channels (the student choice that gave a good trade-off between accuracy and computational cost in the case of ice Ih). To this end, the teacher model is used to label 8000 configurations extracted from classical umbrella sampling trajectories of the dissociation reaction, collected from 16 umbrella windows run for 50 ps each with snapshots stored every 100 fs, and these data are used to train a compact student MACE model with *L* = 0 and 64 channels. As shown in Figure 4, the distilled student reproduces the classical free energy surface of the teacher within statistical uncertainty with nearly a factor of 10 speed-up. As a control, we also consider a directly trained small model on 1000 configurations (same as used to develop the finetuned teacher model) and note that while it results in stable enhanced sampling simulations, it yields a smaller barrier and overstabilizes the dissociated form of water. These results show that transfer learning followed by distillation can transfer the relevant physical knowledge from a high-capacity teacher to a compact student, enabling accurate, robust, and computationally efficient reactive simulations in cases where an equally compact model trained directly on available reference data would be insufficient.

**Figure 4:**
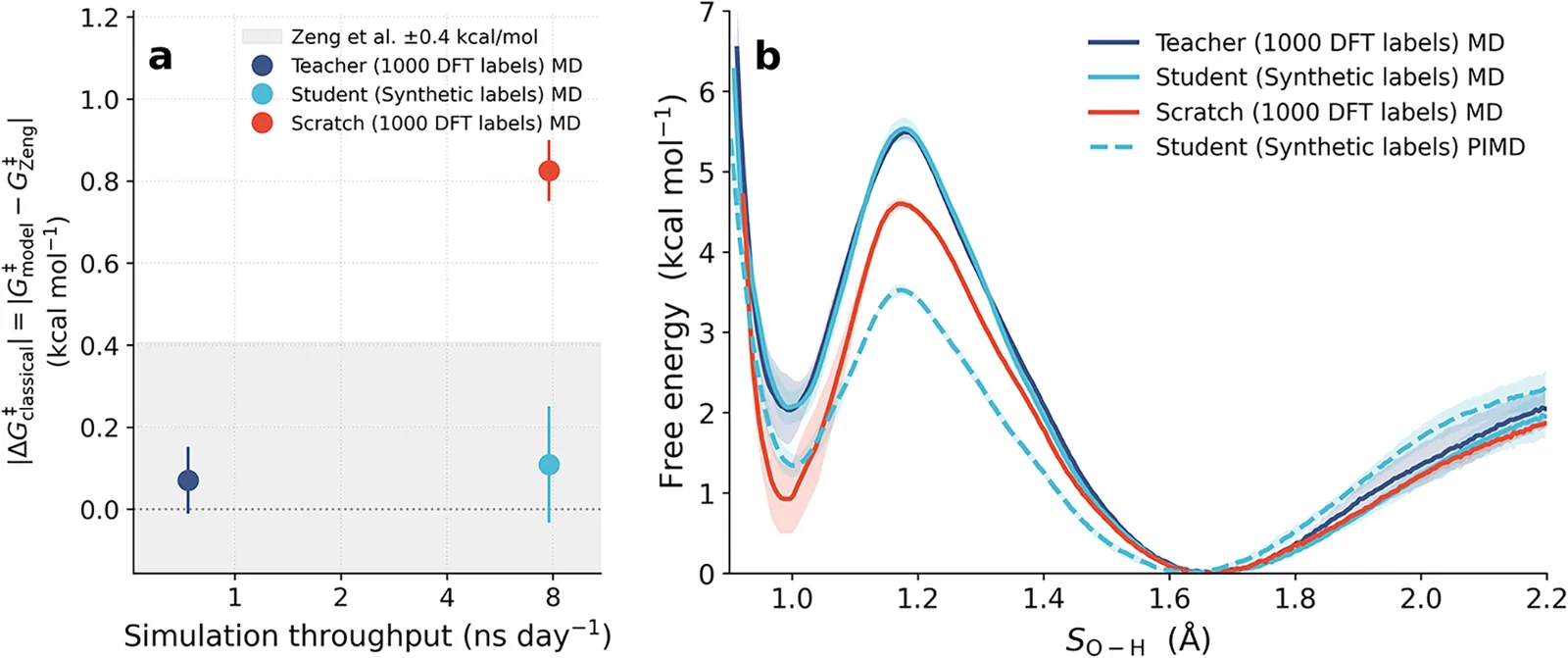
Distillation of the classical free energy profile for water dissociation at the TiO_2_/water interface, and the role of nuclear quantum effects. (a) Speed–accuracy trade-off for the three classical models: the Finetune-1000 teacher (dark blue circle), the classical distilled student (cyan circle), and a small MACE trained from scratch on the same 1000 DFT configurations (orange-red circle). The *y*-axis shows the absolute deviation of the dissociation barrier from the value reported by Zeng et al.,^70^ |Δ*G*_classical_^‡^| = |*G*_model_^‡^ – *G*_Zeng_^‡^|, with *G*_Zeng_^‡^ = 5.43 ± 0.41 kcal/mol. Error bars on each marker show the model’s own block-averaging uncertainty in *G* ^‡^; the gray horizontal band shows the reported uncertainty of *G*_Zeng_^‡^. Throughputs are measured on a single NVIDIA A100-PCIe GPU. (b) Free energy profiles along the umbrella sampling coordinate *S*_O–H_ for the same three classical models and the quantum (PIMD) distilled student (cyan, dashed). Profiles are referenced to the molecularly adsorbed state minimum near *S*_O–H_ ≈ 1.67 Å; the dissociated state lies near *S*_O–H_ ≈ 1 Å. Shaded bands show statistical uncertainty from block averaging. The quantum student is omitted from (a) because its barrier difference reflects nuclear quantum effects rather than a model-accuracy error.

We next investigate whether the same distillation strategy can be extended to quantum nuclei and use this to explore whether NQEs modify the free energy profile of water dissociation at the interface. Obtaining a stable quantum student requires a series of tests of both the training data and the student architecture, which we describe below. As a first test, we used the classical distilled student directly in path-integral molecular dynamics. For the present student architecture, MACE with *L* = 0 and 64 channels, the PIMD trajectory becomes unstable after only a few femtoseconds. This shows that at this model capacity, a student trained only on classical configurations is not transferable to the broader configuration space explored by the quantum nuclei during interfacial proton transfer. We therefore constructed a quantum training set using the teacher. Specifically, we collect 8000 teacher-labeled configurations from 50 ps PIMD umbrella trajectories distributed across the dissociation coordinate and train a new *L* = 0, 64-channel student on these configurations. This model also remains unstable in the PIMD. We next tested whether increasing the expressivity of the student is sufficient to resolve this instability while keeping the quantum training set fixed. For this purpose, we train a more expensive MACE student with *L* = 1 and 64 channels on the same quantum dataset. This model also produces unstable PIMD trajectories. Thus, increasing the expressivity of the student from *L* = 0 to *L* = 1 does not by itself resolve the instability for the training set obtained directly from the short teacher-labeled PIMD trajectories. We therefore next test the second potential limitation, namely, insufficient coverage of the quantum configuration space. To broaden the training distribution, we randomly selected 1600 configurations from the PIMD trajectories and generated 6400 additional structures by rattling them with the *augment-atoms* code,^50,83^ again yielding a total of 8000 teacher-labeled quantum configurations. We first train the original *L* = 0, 64-channel student on this augmented dataset. The resulting model remains unstable in PIMD, showing that broadening the training distribution alone is not sufficient for this compact student architecture. We therefore combined the augmented training set with a more expressive student. Rather than returning to the *L* = 1, 64-channel model considered above, we use *L* = 1 with 32 channels, which provides increased angular expressivity while remaining computationally better suited to the goal of distillation. This model is only marginally more expensive than the original *L* = 0, 64-channel student. A single-point force evaluation timed with ASE on a representative snapshot takes 0.111 s for the *L* = 1, 32-channel model compared to 0.108 s for the *L* = 0, 64-channel model, corresponding to an increase of approximately 3%. The *L* = 1, 32-channel model produces stable umbrella sampling simulations across all windows and is therefore adopted as the final quantum student. Taken together, these tests show that neither the tested increase in model expressivity alone nor broadening the quantum training distribution alone is sufficient to obtain stable PIMD simulations. Stable simulations are obtained when the augmented quantum training set is combined with the more expressive *L* = 1 student.

As shown in Figure 4, the quantum free energy profile exhibits both a lower barrier for the molecular-to-dissociative transformation and a smaller free energy difference between the molecularly adsorbed and dissociated states than the classical profile. NQEs therefore not only soften the barrier region but also stabilize the dissociated state relative to the molecularly adsorbed one. Both the transition state and the dissociated and partially dissociated states involve short O···H configurations in which the proton is shared between a water oxygen and a surface oxygen; quantum delocalization of the proton over these proton-shared bonds redistributes statistical weight from the localized molecular minimum into the bridged transition-state region and into partially dissociated configurations, simultaneously lowering the effective barrier and stabilizing the dissociated side. This delocalization-driven softening of short hydrogen bonds is a generic feature of NQEs in condensed-phase hydrogen-bonded systems.^9,84,85^ Related NQE-driven barrier softening for water dissociation has been reported on stepped Pt(221), Pt(111), and Ru(0001).^86–88^

The computed thermodynamics can be compared with the solid-state ^17^O NMR measurements of Yang et al.,^89^ who quantified surface oxygen speciation on anatase TiO_2_ nanoparticles (≈95% (101)-exposed, ∼20 nm) at ambient temperature (≈298 K) as a function of H_2_^17^O loading. At 3.1–4.7 mmol g^–1^, the populations of nonprotonated bridging O_2c_ and total surface hydroxyl reach a loading-independent plateau, corresponding to Ti_5c_ saturation and additional physisorbed water layers. This high-loading regime is the closest experimental analogue of the flat-slab, bulk-water interface simulated here. Using Yang et al.’s coverage-loading calibration (≈28% Ti_5c_ coverage at 0.3 mmol g^–1^ and ≈46% at 0.5 mmol g^–1^), this regime lies well above Ti_5c_ saturation; each Ti_5c_ site is therefore occupied by either chemisorbed or dissociated water, and the bridging–OH fraction relative to total surface O_2c_ gives the dissociated fraction *P*_diss_. Because the “OH” curve in Figure 3c of that work includes both bridging and terminal hydroxyl ^17^O resonances, and each dissociation event produces one of each, we take $P_{\mathrm{diss}}=\frac{1}{2}\mathrm{OH}$ . Digitizing Figure 3c of ref. 89 at 4.7 mmol g^–1^ gives OH ≈ 0.19 and O_2c_ ≈ 0.90, with $O_{2c}+\frac{1}{2}\mathrm{OH}\approx 1.00$ , corresponding to *P*_diss_ ≈ 10%. Using Δ*G* = −*k*_B_ *T* ln­(*P*_diss_/*P*_mol_) gives Δ*G* ≈ +1.3 kcal mol^–1^ at 298 K.

The PIMD free energy profile was computed at 330 K, following the temperature used for the classical free energy profile reported by Zeng et al.^70^ A direct PIMD calculation at 298 K would require additional computationally demanding umbrella sampling simulations. We therefore estimated the possible effect of the 32 K temperature difference using two approximate tests. First, we applied a first-order entropy correction to the existing 330 K PIMD trajectories, assuming that the entropy difference remains constant over 298–330 K; details are provided in Section S3.D of the SI. This gives a 330-to-298 K shift of +0.34 ± 0.14 kcal mol^–1^ and Δ*G*_PIMD_ = 1.68 ± 0.24 kcal mol^–1^ at 298 K. Because this correction relies on a first-order approximation, we do not interpret it as a precise prediction of the temperature dependence of Δ*G*_PIMD_. As a complementary test of the scale of the temperature effect, classical umbrella sampling gives Δ*G* = 2.1 ± 0.1 kcal mol^–1^ at 300 K and 2.1 ± 0.3 kcal mol^–1^ at 330 K, with unrounded central values differing by only approximately 0.06 kcal mol^–1^. Thus, the two approximate tests indicate temperature effects ranging from negligible to a few tenths of a kcal mol^–1^. Taking the larger +0.34 kcal mol^–1^ correction as a conservative sensitivity estimate, the PIMD value at 298 K remains closer to the NMR-derived value of 1.34 kcal mol^–1^ than the classical result near 2.1 kcal mol^–1^. These estimates remain approximate and do not replace a direct PIMD umbrella sampling calculation at 298 K.

A complementary kinetic isotope signature has been reported by Tan et al.,^90^ who used low-temperature scanning tunneling microscopy to follow the dehydrogenation of single submonolayer water molecules on rutile TiO_2_(110) at 80–100 K, and observed H_2_O dehydrogenation rates 2–4 times faster than D_2_O. They attributed this kinetic isotope effect to the larger zero-point energy of the O–H stretching mode reducing the effective activation barrier. Although the surface termination, water environment, temperature, and the kinetic versus thermodynamic nature of the observable preclude direct comparison with our equilibrium *G* ^‡^, the sign and ZPE-based mechanism of the isotope effect are consistent with the NQE-induced barrier reduction observed in our quantum free energy profile.^91^

An additional source of uncertainty is the finite number of beads used in the PIMD umbrella sampling. Earlier aqueous proton-transfer calculations found a 0.03 kcal mol^–1^ change in a quantum free energy correction between 32 and 64 beads;^92^ in liquid water, the probability-derived free energy contrast between bonded and proton-shared configurations was semiquantitatively converged at 32 beads;^93^ and for the Zundel cation, a position-specific isotope-exchange free energy involving the central shared site differed by approximately 5–6% between 32 and 64 beads.^94^ These indirect benchmarks suggest that the bead-discretization error in the present 32-bead profile may be comparable to its statistical uncertainty, although a system-specific convergence test was not performed.

Separately, to assess foundation-backbone choice for teacher construction, we evaluated MACE-MP-0a­(S) by finetuning it on the same TiO_2_/water datasets containing 500, 1000, 2000, 5000, and 11,667 configurations used for the large-foundation comparison, with 11,667 representing all available nontest DFT-labeled configurations (Figure S13). These models are distinct from the compact MACE control model trained from scratch on 1000 DFT configurations considered above. All finetuned small-foundation models produce stable classical umbrella sampling trajectories across the tested data-size series. The models finetuned on fewer than 5000 DFT configurations show larger deviations from the 11,667-configuration large-foundation model, particularly in the relative stability of the molecular and dissociated states. The 5000-configuration small-foundation model reproduces Δ*G*, *G* ^‡^, and the complete free energy profile with accuracy comparable to the large-foundation models in Figure 3, and the 11,667-configuration small-foundation model confirms this agreement. By comparison, the large-foundation model trained on only 500 configurations already reproduces the large-foundation full-data result to within a fraction of a kcal mol^–1^. Thus, under the present training protocol, the large foundation model reaches comparable accuracy with approximately an order of magnitude fewer DFT-labeled configurations.

A key insight from the primary distillation results is that successful distillation is governed by both training-data coverage and student expressivity, and that these two factors must scale in tandem as the target observable becomes more challenging. For the classical reactive case, the Finetune-1000 teacher transfers cleanly to a compact *L* = 0 student trained on configurations drawn from short teacher-driven MD trajectories across all umbrella-sampling windows. For the quantum problem, the analogous PIMD-trajectory dataset is no longer sufficient on its own: an *L* = 0 student augmented with rattled PIMD configurations is unstable, and an *L* = 1 student trained on PIMD-only configurations without rattling is likewise unstable; only the combination of augmented configurational coverage and *L* = 1 messages yields a PIMD-stable student, which then reveals an NQE-induced reduction of the dissociation barrier of ∼2 kcal/mol relative to the classical profile.

## 3 Conclusions

In this work, we combined transfer learning and knowledge distillation to construct compact MLIPs for three condensed-phase water problems namely, an equilibrium bulk observable to quantum liquid properties and an interfacial reactive free energy profile. For ice Ih, a student distilled from a finetuned MACE teacher is trained only on configurations derived from the low-temperature dataset, but still reproduces the teacher *NPT* density at a higher temperature while being approximately an order of magnitude faster. Models of the same size trained directly on the limited DFT dataset either do not sustain stable MD under these conditions or give larger density errors. For liquid water, a compact ACE student distilled from a Δ-learned CCSD­(T)-quality teacher is also approximately an order of magnitude faster than the teacher, yet reproduces the density isobar, RDFs, diffusion coefficient, and nuclear quantum kinetic energies over 240–370 K. The student captures both the subtle effect of NQEs on the density isobar and the much larger quantum contribution to the hydrogen and oxygen kinetic energies, while retaining agreement with experiment where direct comparisons are available and with MB-pol PIMD benchmarks for the nuclear kinetic energies. For water dissociation at the anatase TiO_2_(101)–water interface, distillation transfers the classical umbrella-sampling free energy profile from a finetuned teacher to a compact student and makes PIMD umbrella sampling practical for incorporating NQEs into the reaction thermodynamics. The resulting quantum free energy profile shows that NQEs change both the barrier and the relative stability of the molecular and dissociated states, bringing the computed reaction thermodynamics into closer agreement with experiment.

The three systems show that the requirements for distillation are not fixed but depend on the observable and on the configuration space sampled during production. For the classical TiO_2_/water free energy profile, a compact *L* = 0, 64-channel MACE student trained on teacher-labeled umbrella sampling configurations reproduces the teacher within statistical uncertainty. To include NQEs at the interface, the same student architecture was not sufficient. The *L* = 0 student remains unstable even when trained on PIMD configurations after additional structures were generated by rattling, whereas an *L* = 1, 32-channel student trained on the augmented quantum dataset supports stable PIMD umbrella sampling. This provides a concrete example in which both the training set and the student architecture must be changed when moving from classical reactive sampling to quantum nuclear sampling.

The TiO_2_/water application also gives a chemical result that would be substantially more expensive to obtain directly with the larger teacher model. The quantum student predicts that NQEs lower the water-dissociation barrier by approximately 2 kcal/mol relative to the classical profile and reduce the molecular–dissociated free energy difference from ≈+2.1 to ≈+1.3 kcal mol^–1^. After applying the first-order temperature correction, the quantum result deviates from the NMR-derived experimental estimate of ≈1.34 kcal mol^–1^ by at most 0.34 ± 0.24 kcal mol^–1^, compared with approximately 0.76 ± 0.10 kcal mol^–1^ for the classical result. This agreement is obtained despite the uncertainties associated with comparing an ideal flat-slab simulation at 330 K to nanoparticle measurements near ambient temperature, and is consistent with nuclear quantum motion stabilizing dissociated or partially dissociated interfacial configurations in this system.

Taken together, these results show that knowledge distillation can make finetuned DFT-quality and Δ-learned CCSD­(T)-quality MLIPs usable in simulations that require demanding sampling methods, including long constant-pressure trajectories, PIMD, and umbrella sampling. We think this opens the door to many problems where accurate potentials must be combined with extensive sampling, especially in catalysis. The additional comparisons involving the size of the foundation MLIPs show that, when a teacher must be constructed by finetuning, the larger MACE-MP-0a­(L) foundation model reaches the required observable accuracy with fewer DFT-labeled configurations than MACE-MP-0a­(S) in both ice Ih and TiO_2_/water. For ice Ih, the large foundation model finetuned on only 50 configurations reproduces the density obtained with the 400-configuration large model at both temperatures and can subsequently be distilled to a 64-channel *L* = 0 model that is faster than the directly finetuned MACE-MP-0a­(S) models. For TiO_2_/water, the large foundation model trained on 500 configurations already reproduces the 11,667-configuration large-foundation result to within a fraction of a kcal mol^–1^, whereas the small foundation model requires approximately 5000 configurations to obtain comparable agreement in Δ*G*, *G* ^‡^, and the complete free energy profile. Direct finetuning of the smaller foundation model is therefore a viable alternative, but under the present training protocols the larger foundation model provides a more data-efficient route to constructing an accurate teacher. Several directions naturally follow from these results. First, the present workflow constructs the distillation dataset in a one-shot manner and then tests whether the student remains stable in the target simulation. Coupling distillation with active learning or uncertainty-guided sampling would make this process more systematic. In particular, a small committee of independently trained compact students could use model disagreement as an uncertainty signal to identify poorly represented configurations and trigger targeted teacher labeling before long production trajectories become unstable.^95–200^ Second, the interface calculations show that test-set errors alone do not determine whether a student will support stable PIMD or enhanced-sampling simulations. A more quantitative connection between the complexity of the sampled configuration space and the minimum required student architecture would make model selection less empirical. Third, the TiO_2_/water teacher was finetuned on classical-nuclei DFT configurations, while the quantum student was trained to cover the additional configurations sampled by PIMD. This strategy is sufficient for the present system, but for reactions where NQEs open qualitatively different pathways, the teacher itself may need to be finetuned or validated on configurations sampled with quantum nuclei. Finally, applying the same approach to other reactive interfaces, proton-coupled processes, solution reactions, and ion-transport problems will test whether the coverage and architecture trends observed here are general across condensed-phase chemistry.

## 4 Methods and Computational Details

### 4.1 Reference Data and Target Systems

For the solid system, ice Ih, we use the dataset of Kaur et al.,^21^ consisting of 500 revPBE-D3(0)^57,98^ -labeled configurations at 100 K and 1 bar, of which 400 are used for training and 100 are reserved as a test set.

For the liquid system, water, the teacher is the baseline+Δ MACE model of O’Neill et al.,^19,99^ which was designed to reproduce CCSD­(T)-quality condensed-phase water behavior through a combination of periodic DFT data and gas-phase CCSD­(T)–DFT energy differences. The corresponding student model is trained on configurations labeled by this teacher.

For the interface system, TiO_2_–water, we use the optB88-vdW^100^ dataset of Zeng et al.^70^ We retain only the TiO_2_–water interface configurations involving pristine and defective anatase and rutile surfaces, since these are the environments relevant to the present TiO_2_–water problem. The resulting dataset contains 12,963 configurations. As described in Section 2, the final benchmark is performed exclusively on the pristine anatase TiO_2_(101)–water interface, while finetuning is carried out on randomly selected subsets drawn from the broader interfacial dataset.

### 4.2 Teacher Models and Student Models

#### 4.2.1 Ice Ih

The teacher model is obtained by finetuning MACE-MP-0a­(L) on the 400-configuration training portion of the 500-configuration dataset of Kaur et al.,^21^ with the remaining 100 configurations used as a test set. For the foundation-backbone comparison, MACE-MP-0a­(S) is finetuned on nested subsets containing 50, 100, 200, and 400 DFT configurations, while MACE-MP-0a­(L) is additionally finetuned on the 50-configuration subset. All finetuning calculations use a random 9:1 training:validation split, energy and force weights of 10, and an initial learning rate of 0.01. Subset finetuning is run for 1000 epochs, with stage two beginning at epoch 750 and a reduced learning rate of 0.00025. When all available data are used, finetuning is instead run for 500 epochs, with stage two beginning at epoch 250. Finetuning is performed with MACE version 0.3.14 using CUDA 12.8.

The MACE students use two interaction layers, correlation order 3, a radial cutoff of 5.0 Å, 10 Bessel radial functions multiplied by a smooth polynomial cutoff, and *l*_max_ = 3. Here, *L* denotes the maximum angular frequency order of the messages in the irreducible-representation basis. The two MACE students considered for ice Ih both use invariant *L* = 0 messages, with 64 and 128 channels, corresponding to 148,944 and 460,432 parameters, respectively. Student training is run for 600 epochs, with stage two beginning at epoch 400.

The ACE student is trained with the Pacemaker package.^101–103^ The model uses a radial cutoff of 6.0 Å, a Finnis–Sinclair-type nonlinear embedding with two atomic properties, up to 1000 basis functions per element, and body orders up to 6. The final model contains 5,296 parameters. Hierarchical fitting is performed with a batch size of 100 and κ = 0.9.

#### 4.2.2 Liquid Water

For liquid water, we use the published baseline + Δ MACE model of O’Neill et al.^19,99^ directly as the teacher. The student is an ACE model trained with the Pacemaker package using a random 9:1 training:validation split. The model uses a radial cutoff of 6.0 Å, a Finnis–Sinclair-type nonlinear embedding with six atomic properties, up to 1000 basis functions per element, and body orders up to 6. The final model contains 14,320 parameters. Hierarchical fitting is performed with a batch size of 100 and κ = 0.9.

#### 4.2.3 TiO_2_/Water Interface

The primary teacher models are obtained by finetuning MACE-MP-0a­(L) on the TiO_2_/water dataset. Ten percent of the available interface configurations are reserved as a strict test set and excluded from all finetuning and student model training. From the remaining configurations, subsets containing 500, 1000, 2000, 5000, and 11,667 structures are used to finetune separate MACE-MP-0a­(L) models. For the foundation-backbone comparison, MACE-MP-0a­(S) is finetuned on the same subsets. All finetuning calculations use a random 9:1 training:validation split, energy and force weights of 10, and an initial learning rate of 0.01. Subset finetuning is run for 1000 epochs, with stage two beginning at epoch 750 and a reduced learning rate of 0.00025. When all available data are used, finetuning is instead run for 500 epochs, with stage two beginning at epoch 250. Finetuning is performed with MACE version 0.3.14 using CUDA 12.8.

The interface MACE students use two interaction layers, correlation order 3, a radial cutoff of 5.0 Å, 10 Bessel radial functions multiplied by a smooth polynomial cutoff, and *l*_max_ = 3. The student architectures considered are *L* = 0 with 64 channels, *L* = 1 with 64 channels, and *L* = 1 with 32 channels. The final quantum student uses *L* = 1 with 32 channels and contains 93,776 parameters. Student training is run for 600 epochs, with stage two beginning at epoch 400.

### 4.3 Molecular Dynamics Simulations

Classical MD simulations with the finetuned MACE-MP-0a­(L) teacher models are performed with LAMMPS^104^ using the ML-IAP package and the mliap pair style. Small and medium MACE student models are interfaced to LAMMPS through the Symmetrix library^32,105,106^ using the symmetrix/mace pair style. ACE simulations are carried out in LAMMPS using the ML-PACE package and the pace pair style. PIMD and TRPMD simulations are performed with i-PI^107,108^ interfaced to LAMMPS.

Unless otherwise stated, classical MD simulations use a timestep of 0.5 fs, and PIMD and TRPMD simulations use a timestep of 0.25 fs. Exceptions for unstable models are reported in Section 2. For classical MD simulations in LAMMPS, a Nosé–Hoover thermostat^109,110^ and barostat are used, with damping times of 100 × d*t* and 1000 × d*t*, respectively, where d*t* is the simulation timestep. For PIMD and TRPMD simulations, the pile_g thermostat^111,112^ with τ = 100 fs and λ = 0.5, together with an isotropic Bussi–Zykova–Parrinello barostat^113^ with τ = 1000 fs for NPT simulations, is used.

For ice Ih, classical NPT simulations are carried out at 100 K and 1 bar and at 220 K and 1 bar. For the original teacher, student, and scratch-model calculations, 5 independent replicas of 1 ns are run after 0.5 ns of equilibration. The additional L-50, S-50–S-400, and L-50-student calculations introduced for the foundation-backbone comparison use 100 ps production trajectories.

For liquid water, classical NPT density calculations are performed from 240 to 370 K in 10 K increments, except that 270 K is replaced by 273.15 K. At each temperature, 5 independent replicas are run until the standard deviation of the density falls below 0.001 g/cm^3^, with a maximum trajectory length ranging from 2 ns at high temperature to 20 ns for supercooled water. Quantum density calculations were performed with PIMD in the NPT ensemble using 32 beads and the same convergence criterion, subject to a maximum trajectory length of 2 ns. RDFs are calculated from classical NVT and PIMD NVT simulations, while self-diffusion coefficients are calculated from classical NVT and TRPMD NVT simulations at the equilibrium densities predicted by the corresponding classical NPT and PIMD NPT simulations. Classical NVT calculations use 5 independent 4 ns replicas after 500 ps of equilibration, while the 32-bead PIMD/TRPMD calculations use 5 independent 0.4 ns replicas after 10 ps of equilibration. For NKE calculations, 64-bead PIMD simulations are performed in the NVT ensemble using 200 ps production trajectories, with the same timestep, thermostat, and other PIMD integration settings as the 32-bead simulations.

For the TiO_2_–water interface, all MD simulations are performed in the NVT ensemble at 330 K using the same simulation cell as in the anatase(101)–water setup of Zeng et al., in order to ensure direct comparability. The simulation volume is fixed such that the water density at the center of the slab is maintained at 1 g/mL. Short unbiased classical MD trajectories of 1 ns are used to analyze interfacial water density profiles and orientational distributions for the untuned and finetuned large models. Short unbiased PIMD simulations of 50 ps are used to compare the final quantum student with the Finetune-1000 teacher. The PIMD NVT settings are the same as those used for liquid water.

### 4.4 Umbrella Sampling for the TiO_2_–Water Interface

The free energy profile for water dissociation on the anatase TiO_2_(101)–water interface is computed by umbrella sampling along the same collective variable used in earlier work on this system.^70,81^ The collective variable *S*_O–H_ is a soft-min approximation to the minimum distance between a particular surface oxygen atom and any hydrogen atom in the system

1 $$S_{O\text{−}H}=\frac{\lambda }{\ln\left[\sum_{i}\exp\left(\lambda /r_{iO}\right)\right]},,\lambda =500\;Å$$

In eq 1, the index *i* runs over all hydrogen atoms, *r*_*i*O_ is the distance from the *i*-th hydrogen to the chosen surface oxygen, and the large *λ* ensures that *S*_O–H_ is dominated by the smallest *r*_*i*O_. Values of *S*_O–H_ ≈ 1 Å correspond to the dissociated state, where a proton has bonded directly to the surface oxygen, while *S*_O–H_ ≈ 1.67 Å corresponds to the molecularly adsorbed state, where the closest hydrogen belongs to the water molecule sitting above the surface oxygen.

The umbrella centers span 0.96 to 2.10 Å, and the force constants range from 125 to 800 kcal/mol. Each window is equilibrated for 20 ps before a production run of 1 ns for the teacher model and quantum student, and 2 ns for the classical student. PIMD umbrella sampling uses 32 beads. Free energy profiles are estimated using WHAM.^114,115^ Statistical uncertainties are estimated by block averaging: each trajectory is divided into three equal parts, three free energy profiles are reconstructed independently, and the standard deviation among them is used as the uncertainty estimate.

## Supplementary Material



## Data Availability

The training datasets, trained models, and simulation input files used in this work are available at https://github.com/Alexandrina-Chen/data-for-mlip-kd.

## Notes

Ice Ih energy and force validation and foundation-backbone comparisons; liquid water radial distribution functions, densities, diffusion coefficients, nuclear kinetic energies, and convergence tests; and TiO_2_/water teacher validation, interfacial structure, foundation-backbone comparisons, and temperature correction to the water-dissociation free energy (PDF)
