# Supplementary material for: Distilling First-Principles Accuracy into Compact Machine Learning Potentials for Condensed-Phase Chemistry
Source: J Chem Theory Comput. 2026 Oct 9;22(20):10928–42. doi: 10.1021/acs.jctc.6c01131 (PMC13718552; doi:10.1021/acs.jctc.6c01131)
Supplement: Supplementary file 1 [file ct6c01131_si_001.pdf]

Supporting Information for "Distilling first-principles accuracy into compact machine learning potentials for condensed-phase chemistry"

Sijia Chen,[1, a)] Niamh O'Neill,[2] Benjamin X. Shi,[1] and Venkat Kapil[3, 4, b)]

[1)] *Initiative for Computational Catalysis, Flatiron Institute, New York, New York 10010, United States*

[2)] *Max Planck Institute for Polymer Research, Mainz 55128, Germany*

[3)] *Department of Physics and Astronomy, University College London, 7-19 Gordon St, London WC1H 0AH, UK*

[4)] *Thomas Young Centre and London Centre for Nanotechnology, 9 Gordon St, London WC1H 0AH, UK*

[a)] Electronic mail: schen@flatironinstitute.org

[b)] Electronic mail: v.kapil@ucl.ac.uk

CONTENTS

S1. Additional Information for Solid System S3

A. Energy validation S3

B. Force validation S5

C. Foundation-backbone comparison for ice Ih teacher construction S7

S2. Additional Information for Liquid System S9

A. Liquid Water Radial Distribution Functions at 300 K: Teacher, Student, and Experiment S9

B. Liquid Water Density S10

C. Fixed-budget low-temperature enrichment of the student training set S11

D. Experimental liquid densities of H_2O and D_2O S13

E. Self-Diffusion Coefficient and Finite-Size Correction S14

F. Nuclear Quantum Kinetic Energy S16

S3. Additional Information for Interface System S18

A. Finetuned Teacher Model Validation on the DFT Test Set S18

B. Classical Interfacial Water Structure S23

C. Foundation-backbone comparison for TiO_2/water teacher construction S24

D. Estimated temperature correction to the PIMD interfacial water-dissociation free energy S26

References S27

S1. ADDITIONAL INFORMATION FOR SOLID SYSTEM

A. Energy validation

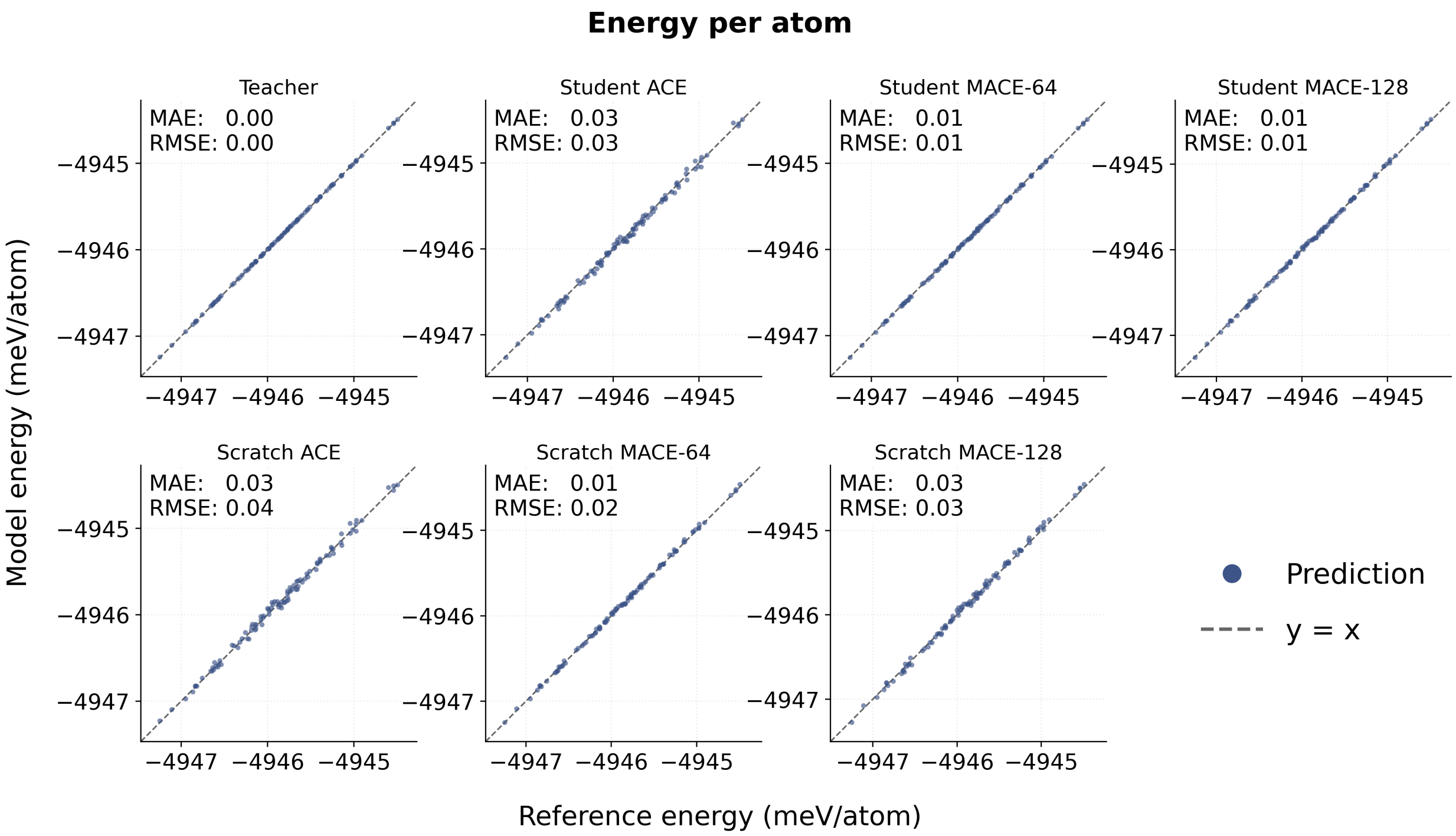

FIG. S1. Parity plot of predicted energy per atom by different machine learning interatomic potentials (MLIPs) against reference revPBE-D3(0) density functional theory (DFT) energies for the solid (ice Ih) system on 100 test-set configurations.

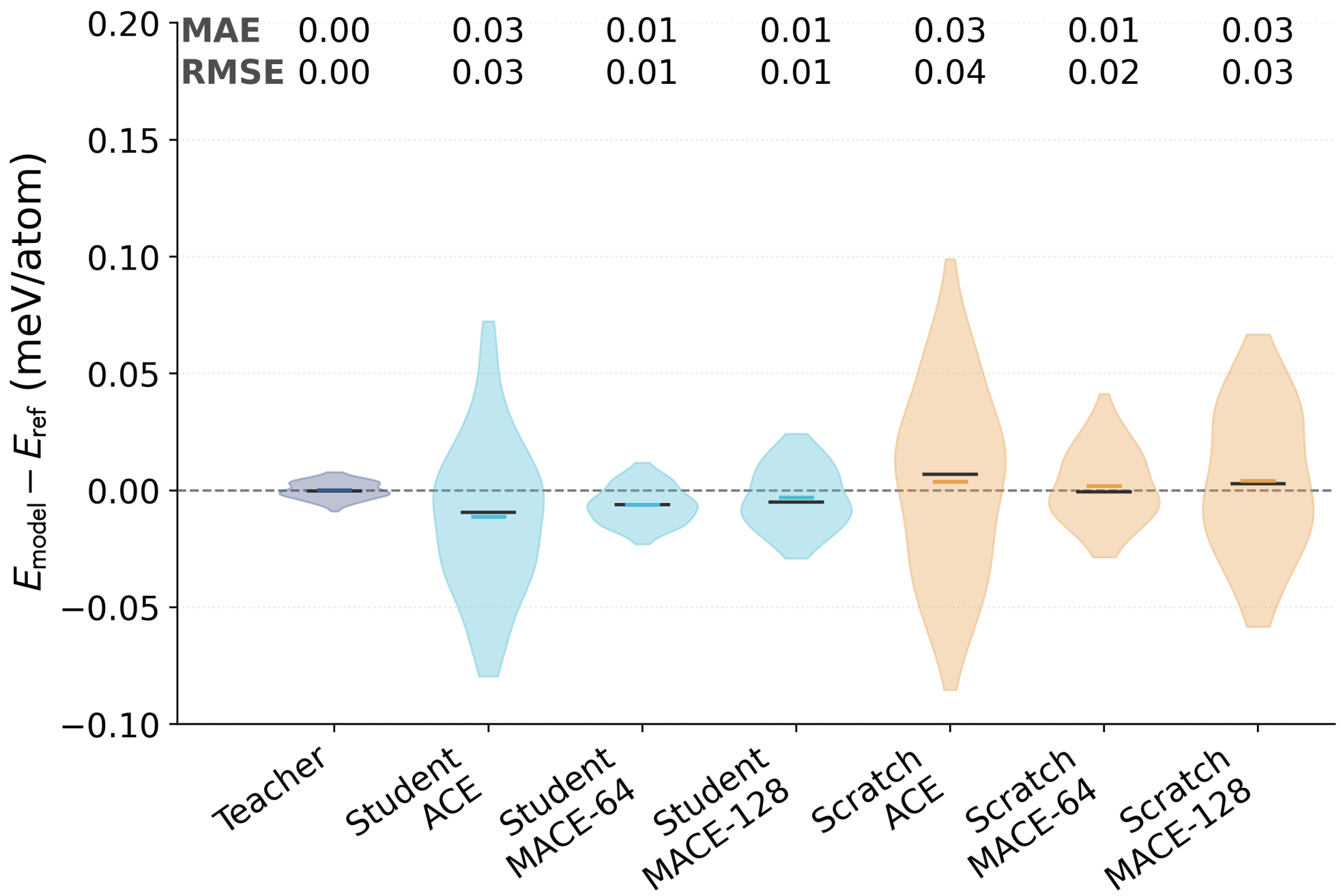

FIG. S2. Violin plots of the energy per atom difference between different machine learning interatomic potentials and reference revPBE-D3(0) density functional theory (DFT) for the solid (ice Ih) system on 100 test set configurations.

B. Force validation

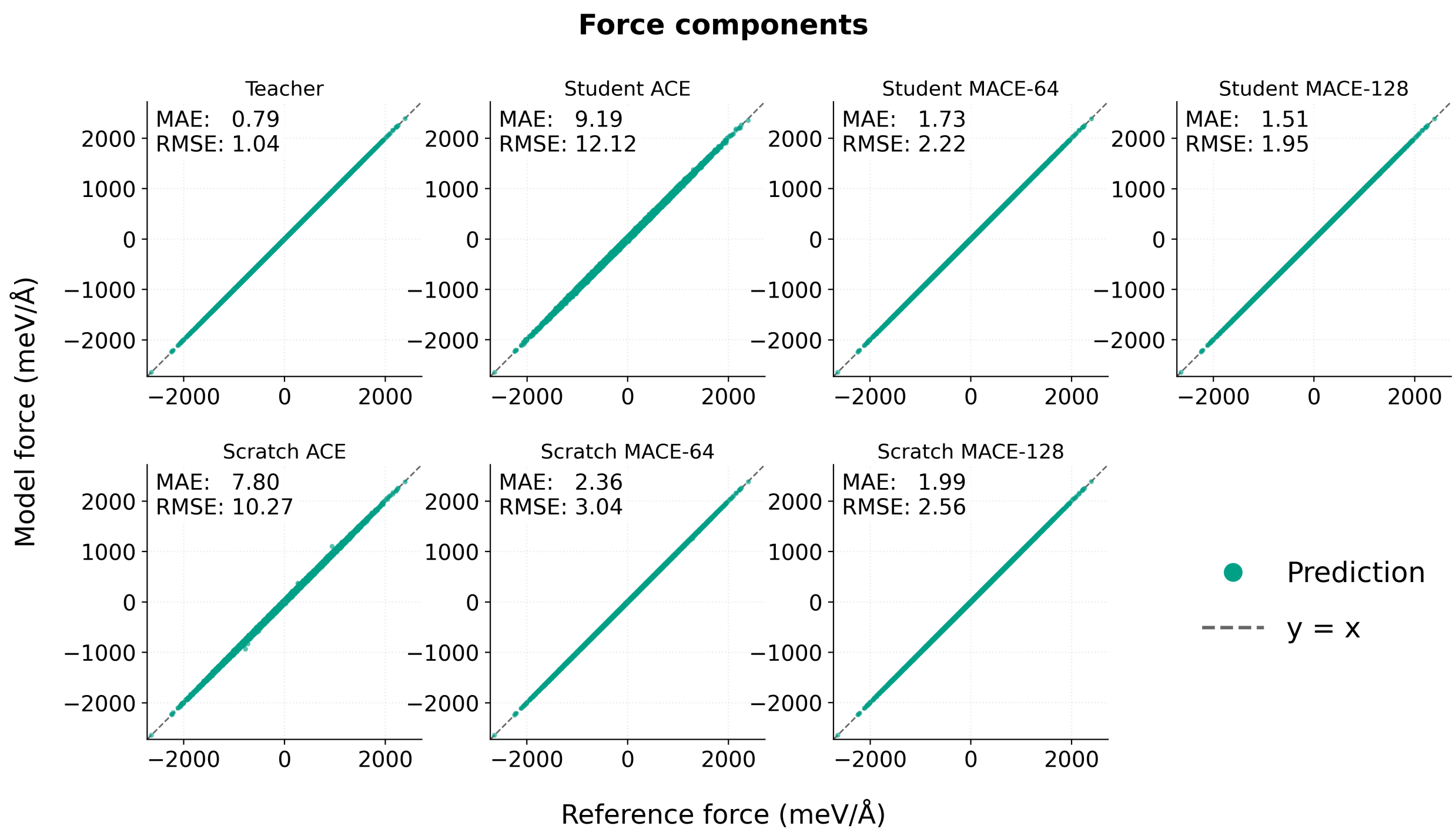

FIG. S3. Parity plot of predicted forces by different machine learning interatomic potentials (MLIPs) against reference revPBE-D3(0) density functional theory (DFT) forces for the solid (ice Ih) system on 100 test-set configurations.

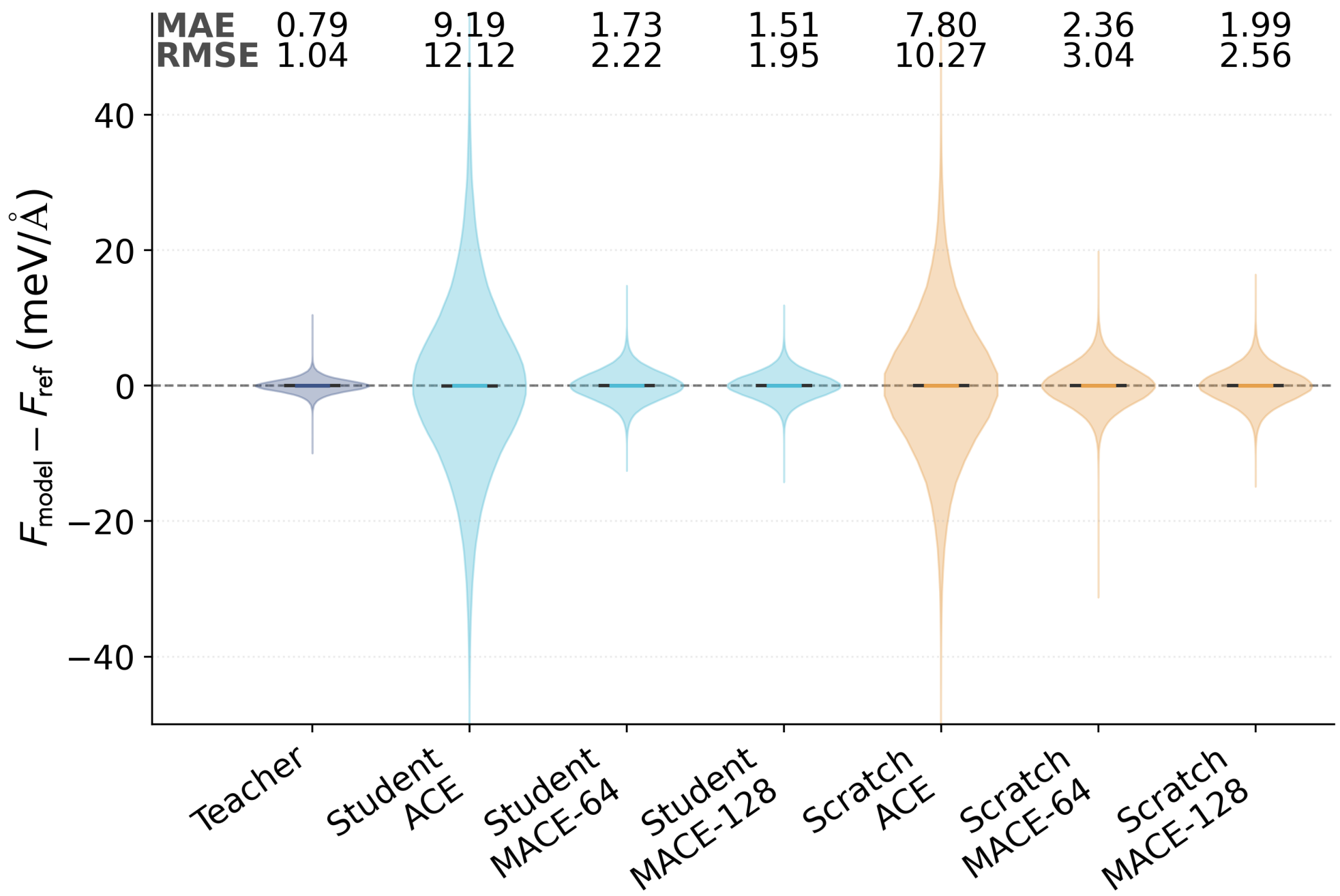

FIG. S4. Violin plots of the force component differences between different machine learning interatomic potentials (MLIPs) and reference revPBE-D3(0) density functional theory (DFT) for the solid (ice Ih) system on 100 test set configurations.

C. Foundation-backbone comparison for ice Ih teacher construction

For S-100, S-200, and S-400, the reported results combine three independent finetuning runs and five production replicas per run. S-50 was evaluated using the single available finetuning run and five production replicas. The reported density uncertainty for S-100, S-200, and S-400 is the sample standard deviation across the 15 training-seed/production-replica density estimates; for all other models, it is the sample standard deviation across five production replicas. Figure S5 reports the signed density error relative to L-400, as defined in Eq. (S1):

$$\Delta\rho = \rho_{\mathrm{model}} - \rho_{\mathrm{L\text{-}400}}. \tag{S1}$$

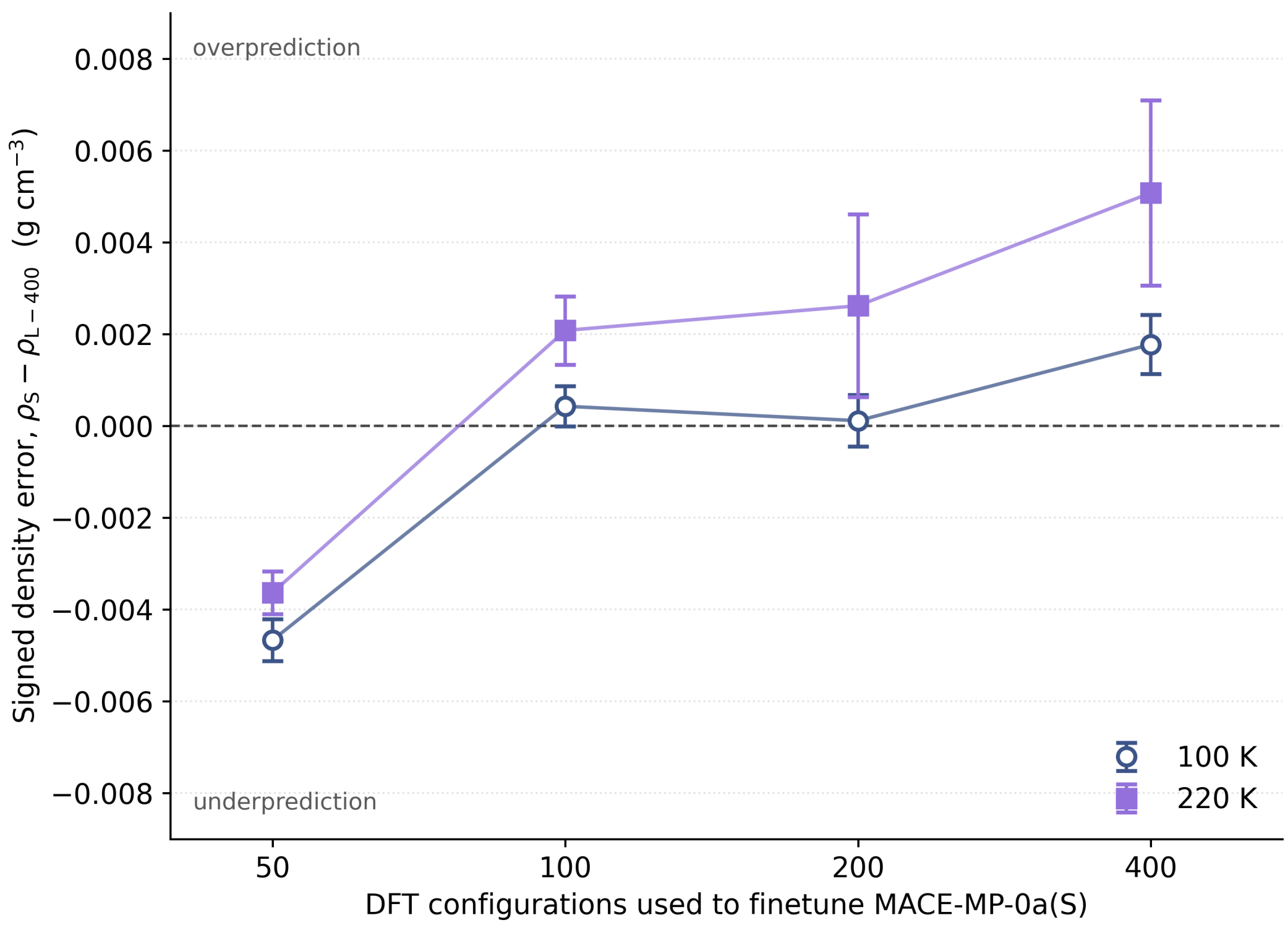

FIG. S5. Signed ice Ih density error relative to L-400 at 100 and 220 K. S-50, S-100, S-200, and S-400 are MACE-MP-0a(S) models finetuned directly on the indicated numbers of DFT configurations. S-100, S-200, and S-400 are averaged over three independent finetuning runs and five production replicas per run; S-50 uses one finetuning run and five production replicas. Error bars combine the corresponding model standard deviation with the standard deviation of the five L-400 replicas in quadrature. The horizontal line marks the L-400 reference.

TABLE S1. Classical NPT densities of ice Ih obtained with the L-400 and L-50 teachers, their distilled students, and the directly finetuned MACE-MP-0a(S) series. Values in parentheses are sample standard deviations in the last reported digit(s).

Model	Training data	100 K	220 K
L-400 teacher	400 DFT	0.9223(2)	0.898(0)
ACE student	2,000 L-400 labels	0.930(0)	0.919(0)†
MACE, $L = 0$, 64 channels	2,000 L-400 labels	0.923(0)	0.901(0)
MACE, $L = 0$, 128 channels	2,000 L-400 labels	0.920(0)	0.897(0)
L-50 teacher	50 DFT	0.922(0)	0.900(0)
MACE, $L = 0$, 64 channels	2,000 L-50 labels	0.920(0)	0.897(1)
MACE, $L = 0$, 128 channels	2,000 L-50 labels	0.922(1)	0.899(1)
S-50	50 DFT	0.917(0)	0.894(0)
S-100	100 DFT	0.923(0)	0.900(1)
S-200	200 DFT	0.922(1)	0.901(2)
S-400	400 DFT	0.924(1)	0.903(2)

Densities are in g cm^{-3}. †Stable only with a 0.25 fs timestep and categorized as unstable at the standard 0.5 fs timestep in main-text Fig. 1.

S2. ADDITIONAL INFORMATION FOR LIQUID SYSTEM

A. Liquid Water Radial Distribution Functions at 300 K: Teacher, Student, and Experiment

Comparison with experiment highlights the role of nuclear quantum effects (NQEs). The classical RDFs are somewhat more structured than the experimental curves. Including NQEs via PIMD substantially improves agreement: the O–O first peak height decreases from 2.74 (classical) to 2.61 (PIMD), closer to the experimental value of 2.50 ± 0.06,[1] while the peak width changes only slightly. In the O–H RDF, the intramolecular peak near 1.0 Å becomes broader and lower, reflecting quantum broadening of the O–H bond-length distribution due to zero-point motion; the intermolecular peaks are also slightly reduced.

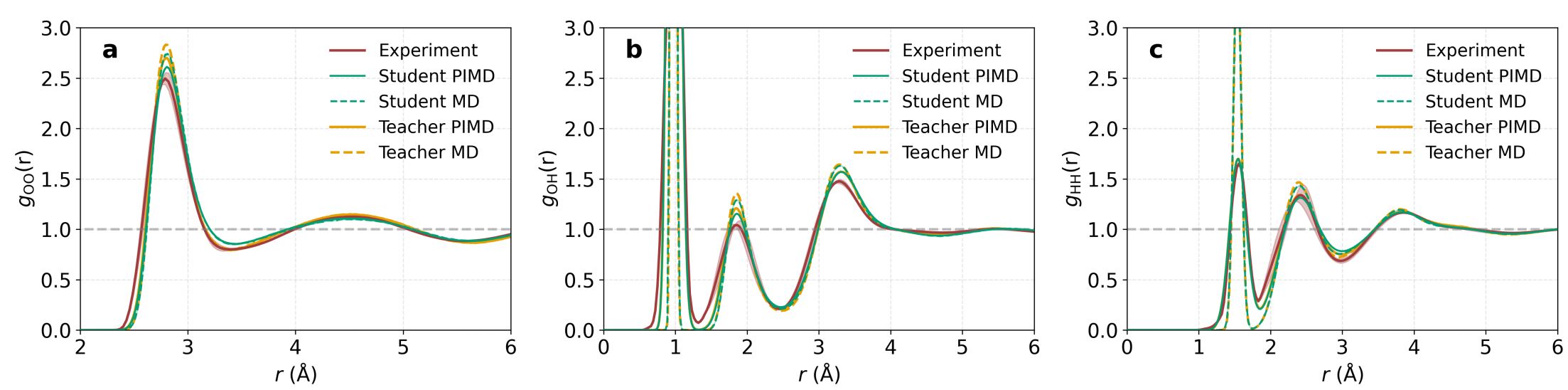

FIG. S6. (a) O–O, (b) O–H, and (c) H–H radial distribution functions of liquid water at 300 K predicted by the student and teacher models with classical molecular dynamics (MD) and path-integral molecular dynamics (PIMD), compared with the experimental data of Soper.[1,2] Experimental intermolecular RDFs are from Ref.;[1] intramolecular contributions are digitized from Ref.[2] and merged with the intermolecular data at their intersection.

B. Liquid Water Density

TABLE S2. Equilibrium density of liquid water from classical NPT simulations with the student model at 1 bar.

Temperature (K)	Density (g cm^{-3})	Std (g cm^{-3})
240.00	0.9944	0.0007
250.00	1.0019	0.0010
260.00	1.0049	0.0005
273.15	1.0054	0.0009
280.00	1.0042	0.0002
290.00	1.0010	0.0008
300.00	0.9980	0.0009
310.00	0.9929	0.0007
320.00	0.9884	0.0010
330.00	0.9823	0.0008
340.00	0.9767	0.0009
350.00	0.9704	0.0004
360.00	0.9646	0.0008
370.00	0.9572	0.0009

TABLE S3. Equilibrium density of liquid water from PIMD NPT simulations (32 beads) with the student model at 1 bar.

Temperature (K)	Density (g cm^{-3})	Std (g cm^{-3})
240.00	0.9962	0.0032
250.00	1.0013	0.0042
260.00	1.0034	0.0017
273.15	1.0017	0.0013
280.00	1.0015	0.0020
290.00	0.9976	0.0009
300.00	0.9947	0.0010
310.00	0.9902	0.0009
320.00	0.9850	0.0005
330.00	0.9806	0.0010
340.00	0.9755	0.0009
350.00	0.9692	0.0007
360.00	0.9632	0.0009
370.00	0.9566	0.0006

C. Fixed-budget low-temperature enrichment of the student training set

For the low-temperature-enriched student, classical NPT densities were evaluated at 1 bar using five independent 5 ns production trajectories at each temperature. Figure S7 and Table S4 report the resulting density isobar and numerical values.

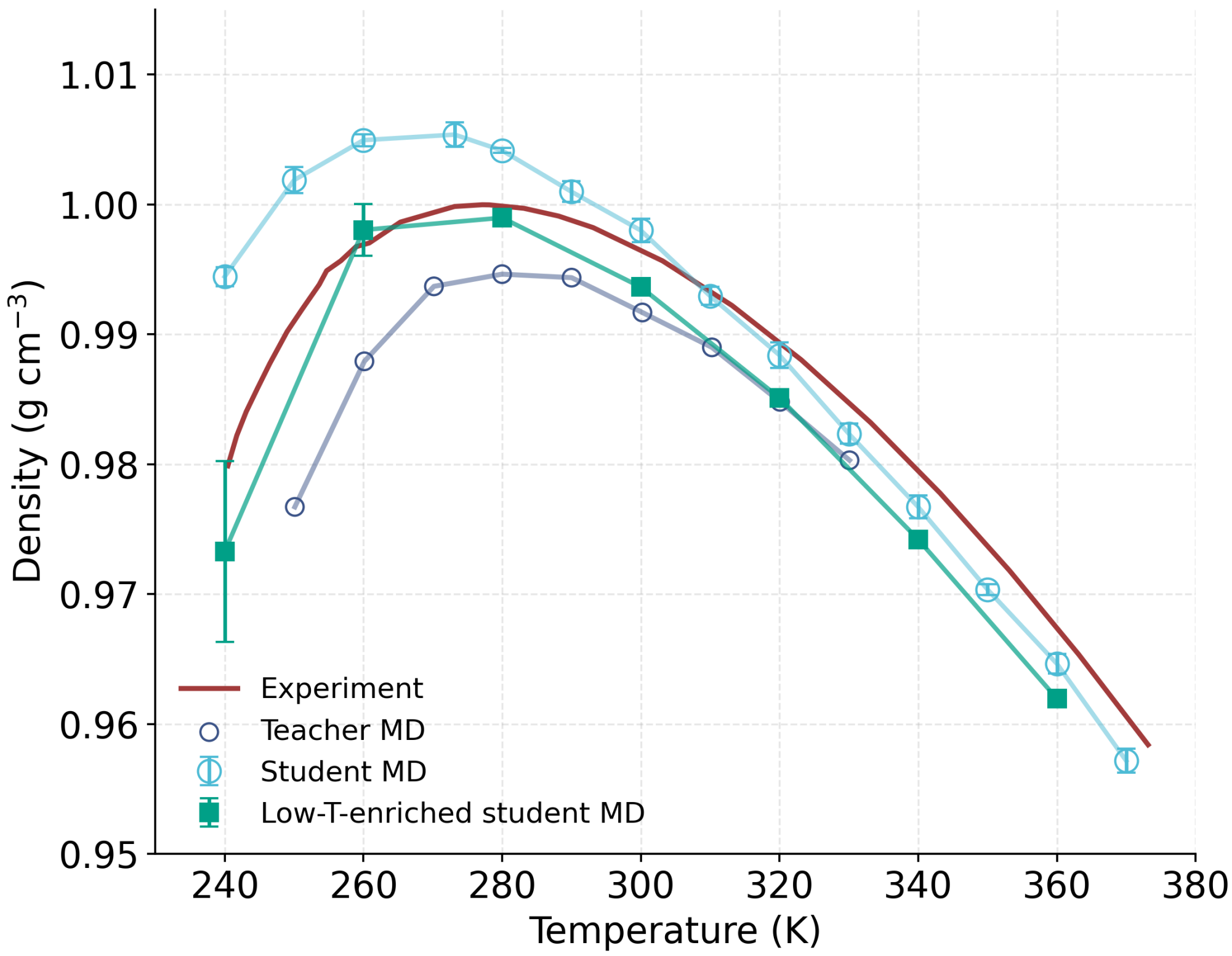

FIG. S7. Classical liquid-water density at 1 bar predicted by the initial student and the low-temperature-enriched student, compared with the teacher model of O'Neill *et al.*[3] and experiment.[4–6] The student error bars show the sample standard deviation across five independent replicas. Lines connecting simulation points are guides to the eye.

TABLE S4. Classical liquid-water densities at 1 bar from the initial and low-temperature-enriched students. Each density is first averaged over its production trajectory; the reported mean and sample standard deviation are then calculated across five independent replicas. All densities are in g cm^{-3}.

Temperature (K)	Initial student	Std	Low-T-enriched student	Std
240	0.9944	0.0007	0.9733	0.0070
260	1.0049	0.0005	0.9980	0.0020
280	1.0042	0.0002	0.9990	0.0003
300	0.9980	0.0009	0.9936	0.0005
320	0.9884	0.0010	0.9851	0.0004
340	0.9767	0.0009	0.9742	0.0004
360	0.9646	0.0008	0.9620	0.0006

D. Experimental liquid densities of H_2O and D_2O

Liquid densities at ambient pressure were compiled from experimental measurements of H_2O and D_2O. For H_2O, supercooled-liquid densities were taken from Hare and Sorensen,[7] while stable-liquid densities were taken from Kell and Whalley[8] and Gildseth et al..[9] For D_2O, densities were taken from Blahut et al.[10] in the cold/supercooled range, and from Millero et al.[11] and Chang and Tung[12] in the stable-liquid range. The D_2O densities were rescaled by the molar-mass ratio $M_{H_2O}/M_{D_2O} = 0.899526$ before comparison with H_2O. The difference reported in the final column is defined in Eq. (S2):

$$\Delta\rho = \rho_{H_2O} - \rho_{D_2O}\frac{M_{H_2O}}{M_{D_2O}}. \tag{S2}$$

All tabulated values are rounded to four decimal places and are reported in g cm^{-3}. No D_2O values are reported at 240 and 250 K because the experimental D_2O data used here do not extend below 254 K.

TABLE S5. Ambient-pressure liquid densities of H_2O and D_2O. The D_2O densities have been rescaled by M_{H_2O}/M_{D_2O} before comparison with H_2O. The difference is defined as $\Delta\rho = \rho_{H_2O} - \rho_{D_2O}M_{H_2O}/M_{D_2O}$.

T / K	ρ_{H_2O} / g cm^{-3}	$\rho_{D_2O}M_{H_2O}/M_{D_2O}$ / g cm^{-3}	$\Delta\rho$ / g cm^{-3}
240	0.9791	—	—
250	0.9910	—	—
260	0.9970	0.9881	0.0089
270	0.9997	0.9929	0.0068
280	1.0001	0.9948	0.0053
290	0.9990	0.9947	0.0043
300	0.9967	0.9931	0.0036
310	0.9933	0.9904	0.0029
320	0.9893	0.9868	0.0025
330	0.9845	0.9824	0.0021
340	0.9792	0.9773	0.0019
350	0.9735	0.9716	0.0019
360	0.9674	0.9654	0.0020
370	0.9609	0.9586	0.0023

E. Self-Diffusion Coefficient and Finite-Size Correction

Self-diffusion coefficients obtained from MD simulations with periodic boundary conditions are subject to a well-known finite-size artifact arising from hydrodynamic interactions between periodic images.[13] We correct for this effect using the Yeh–Hummer formula:

$$D_{\text{corrected}} = D_{\text{PBC}} + \frac{k_B T\,\xi}{6\pi\,\eta(T)\,L} \tag{S3}$$

where D_{PBC} is the diffusion coefficient computed directly from the simulation with periodic boundary conditions, k_B is the Boltzmann constant, T is the temperature, $\xi = 2.837297$ is a dimensionless constant for cubic simulation cells, $\eta(T)$ is the shear viscosity of water at temperature T, and L is the cubic box length.

We use the experimental shear viscosity of water at each simulation temperature for the finite-size correction. For temperatures at or above 253.15 K, we use the IAPWS 2008 formulation of Huber *et al.*[14] For temperatures below 253.15 K (i.e., 240 K and 250 K in this work), where the IAPWS formulation is not defined, we use the power-law fit of Dehaoui *et al.*,[15] which provides viscosity data for deeply supercooled water.

Tables S6 and S7 report both the uncorrected and finite-size-corrected diffusion coefficients from classical MD and TRPMD simulations, respectively. These data correspond to Figure 2c of the main text.

TABLE S6. Self-diffusion coefficient of liquid water from classical NVT simulations with the student model, before and after the Yeh–Hummer finite-size correction (Eq. S3).

Temperature (K)	$D_{\text{corrected}}$ (Å^2/ps)	Std (Å^2/ps)	D_{PBC} (Å^2/ps)
240.00	0.0229	0.0008	0.0204
250.00	0.0431	0.0010	0.0369
260.00	0.0694	0.0011	0.0580
273.15	0.1165	0.0024	0.0961
280.00	0.1454	0.0015	0.1193
290.00	0.1917	0.0011	0.1559
300.00	0.2476	0.0022	0.2007
310.00	0.3101	0.0025	0.2505
320.00	0.3817	0.0030	0.3079
330.00	0.4533	0.0032	0.3638
340.00	0.5410	0.0055	0.4341
350.00	0.6334	0.0052	0.5078
360.00	0.7331	0.0041	0.5873
370.00	0.8238	0.0050	0.6566

TABLE S7. Self-diffusion coefficient of liquid water from TRPMD NVT simulations (32 beads) with the student model, before and after the Yeh–Hummer finite-size correction (Eq. S3).

Temperature (K)	$D_{\text{corrected}}$ (Å^2/ps)	Std (Å^2/ps)	D_{PBC} (Å^2/ps)
240.00	0.0347	0.0052	0.0322
250.00	0.0528	0.0047	0.0466
260.00	0.0794	0.0039	0.0681
273.15	0.1276	0.0044	0.1072
280.00	0.1562	0.0056	0.1301
290.00	0.2024	0.0024	0.1667
300.00	0.2481	0.0110	0.2013
310.00	0.3120	0.0072	0.2525
320.00	0.3918	0.0119	0.3181
330.00	0.4492	0.0064	0.3597
340.00	0.5257	0.0173	0.4189
350.00	0.6056	0.0169	0.4801
360.00	0.7095	0.0246	0.5638
370.00	0.8065	0.0228	0.6393

F. Nuclear Quantum Kinetic Energy

The per-atom quantum nuclear kinetic energy tensor was evaluated using the centroid-virial estimator[16] and decomposed in a local molecular frame following the convention of Cheng *et al.*[17] For each hydrogen atom, $\hat{e}_1$ is directed along its O–H bond (stretch), $\hat{e}_2$ lies in the HOH plane perpendicular to $\hat{e}_1$ (in-plane bend/libration), and $\hat{e}_3$ is normal to the HOH plane (out-of-plane libration). For oxygen, $\hat{e}_1$ lies along the HOH bisector (C_2 axis), $\hat{e}_2$ is perpendicular to the bisector within the molecular plane (antisymmetric direction), and $\hat{e}_3$ is the molecular-plane normal. The diagonal elements of the rotated, ensemble-averaged tensor yield three directional components per species; their trace gives the total $\langle E_{\mathrm{K}} \rangle$. All simulation parameters were identical to those described in the Methods section, except that $P = 32$ and $P = 64$ ring-polymer beads were compared to assess convergence. Production trajectories of 200 ps with $P = 64$ were collected in the NVT ensemble at each temperature; statistical uncertainties were estimated as block-averaged standard errors over five equal blocks.

To assess bead convergence, we compared $P = 32$ and $P = 64$ results at 273.15 K from independent equilibrations (Table S8). The $P = 32 \rightarrow 64$ difference in the hydrogen total is 3.6 meV, concentrated almost entirely in the O–H stretch component (+3.3 meV), which is the highest-frequency intramolecular mode and therefore the most demanding to converge. The remaining hydrogen components and all oxygen components change by less than 0.3 meV. We also compare with MB-pol PIMD results at 273 K from Cheng *et al.*;[17] the $P = 64$ student values agree with MB-pol with $P = 64$ to within 0.2 meV on every component.

TABLE S8. Bead convergence of the molecular-frame kinetic energy tensor components and comparison with MB-pol at 273 K. All values in meV. MB-pol values are read from Figure 2 of Cheng *et al.*[17] Uncertainties on the student values are block-averaged standard errors (5 blocks).

Component	$P = 32$	$P = 64$	MB-pol
H stretch	99.10 ± 0.03	102.39 ± 0.04	102.6
H in-plane	33.07 ± 0.02	33.26 ± 0.02	33.3
H out-of-plane	21.46 ± 0.03	21.57 ± 0.04	21.4
H total	153.63 ± 0.04	157.23 ± 0.06	157.2
O bisector	18.74 ± 0.01	18.90 ± 0.01	18.8
O antisymmetric	19.86 ± 0.01	20.15 ± 0.01	20.2
O out-of-plane	12.95 ± 0.01	12.96 ± 0.01	12.9
O total	51.55 ± 0.01	52.00 ± 0.01	52.0

TABLE S9. Temperature dependence of the per-atom quantum nuclear kinetic energy for hydrogen and oxygen in liquid water computed with $P = 64$ PIMD. Uncertainties are block-averaged standard errors (5 blocks). The classical equipartition value $\frac{3}{2}k_{\mathrm{B}}T$ is listed for reference.

T (K)	$\frac{3}{2}k_{\mathrm{B}}T$ (meV)	$\langle E_{\mathrm{K}}(\mathrm{H})\rangle$ (meV)	$\langle E_{\mathrm{K}}(\mathrm{O})\rangle$ (meV)
240.00	31.03	156.15 ± 0.06	48.44 ± 0.01
250.00	32.32	156.43 ± 0.08	49.48 ± 0.01
260.00	33.62	156.80 ± 0.04	50.58 ± 0.01
273.15	35.32	157.23 ± 0.06	52.00 ± 0.01
280.00	36.20	157.49 ± 0.03	52.75 ± 0.01
290.00	37.50	157.88 ± 0.03	53.85 ± 0.01
300.00	38.79	158.29 ± 0.01	54.96 ± 0.01
310.00	40.08	158.68 ± 0.04	56.08 ± 0.01
320.00	41.38	159.10 ± 0.03	57.20 ± 0.01
330.00	42.67	159.54 ± 0.01	58.31 ± 0.01
340.00	43.97	160.02 ± 0.02	59.45 ± 0.00
350.00	45.26	160.50 ± 0.02	60.59 ± 0.01
360.00	46.55	161.01 ± 0.03	61.73 ± 0.01
370.00	47.85	161.50 ± 0.02	62.87 ± 0.01

S3. ADDITIONAL INFORMATION FOR INTERFACE SYSTEM

A. Finetuned Teacher Model Validation on the DFT Test Set

Element-resolved force parity plots for the finetuned large MACE models and the untuned MACE-MP-0 foundation model[18] against optB88-vdW[19] DFT reference forces are shown in Figures S8–S11.

The overall force RMSE decreases monotonically from 65.54 meV/Å (Finetune-500) to 46.78 meV/Å (Finetune-all) as the finetuning set grows, whereas the untuned foundation model exhibits a substantially larger RMSE of 134.63 meV/Å (Figure S8). Decomposing by element reveals that the accuracy improvement with additional data is most pronounced for hydrogen (RMSE from 35.92 to 22.14 meV/Å) and titanium (from 88.65 to 48.21 meV/Å), while oxygen forces converge more quickly (from 75.59 to 59.53 meV/Å) (Figures S9–S11). The untuned model performs markedly worse on all three elements, with Ti forces showing the largest degradation (RMSE 230.09 meV/Å). These results confirm that finetuning on even a modest number of configurations (e.g., 500) substantially improves force accuracy relative to the untuned foundation model, and that additional data continues to yield incremental gains.

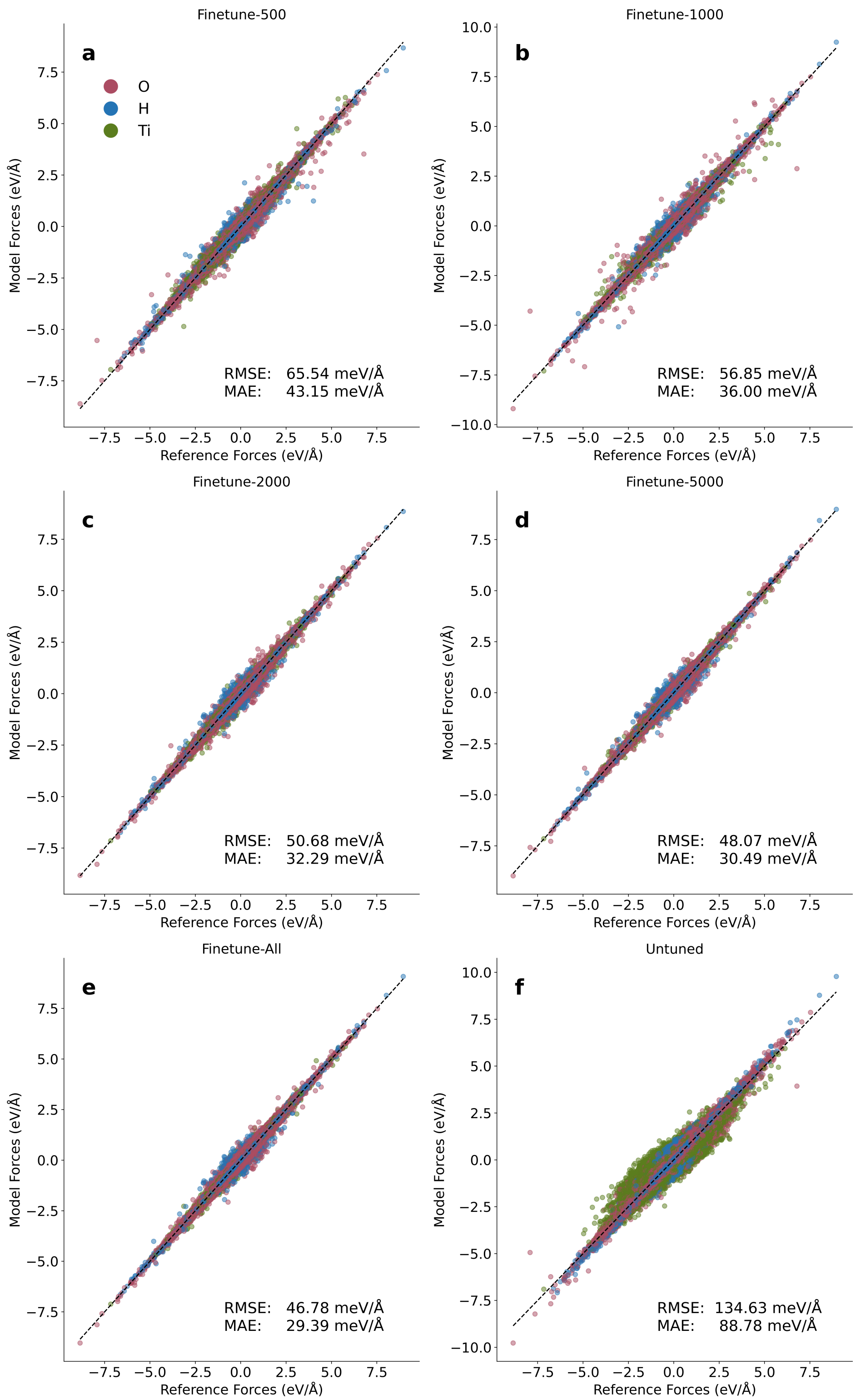

FIG. S8. Force parity plots for all atom types on the DFT hold-out test set. Panels (a)–(e) show finetuned models with increasing training set sizes (Finetune-500, 1000, 2000, 5000, and all), and panel (f) shows the untuned MACE-MP-0 foundation model. Reference forces are from optB88-vdW DFT. Colors indicate element type: O (red), H (blue), Ti (green).

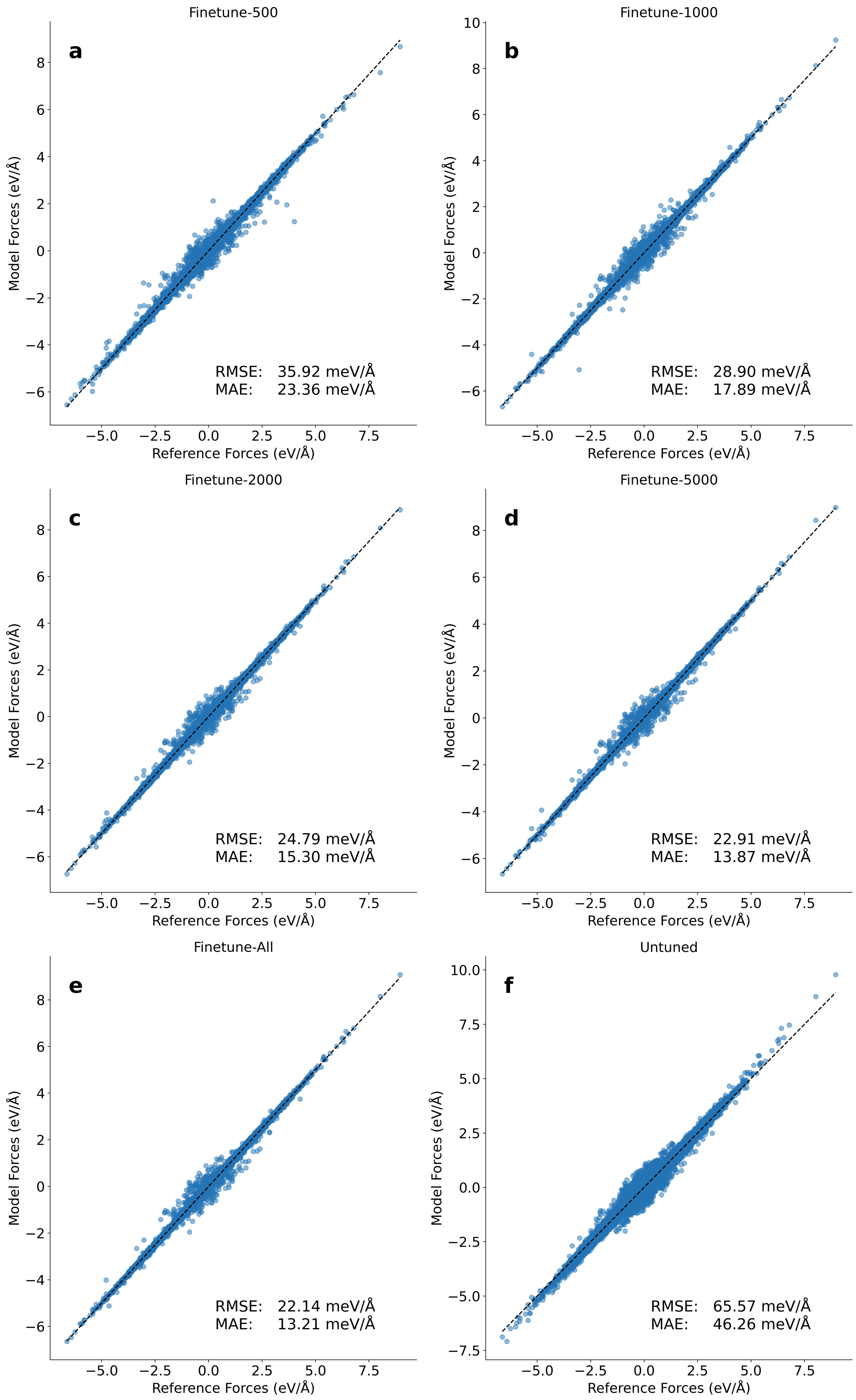

FIG. S9. Force parity plots for hydrogen atoms on the DFT hold-out test set. Panels (a)–(e) show finetuned models with increasing training set sizes, and panel (f) shows the untuned foundation model.

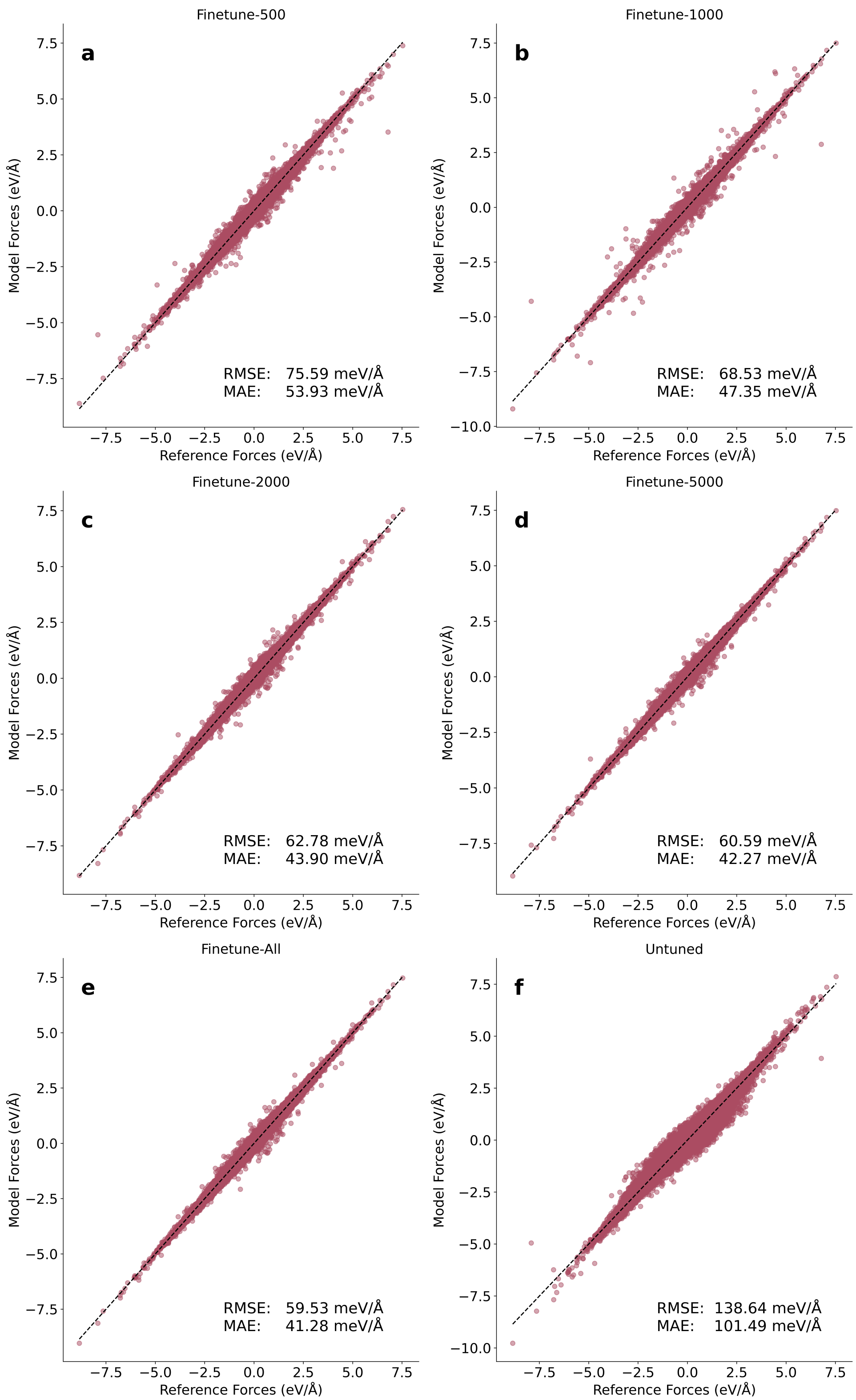

FIG. S10. Force parity plots for oxygen atoms on the DFT hold-out test set. Panels (a)–(e) show finetuned models with increasing training set sizes, and panel (f) shows the untuned foundation model.

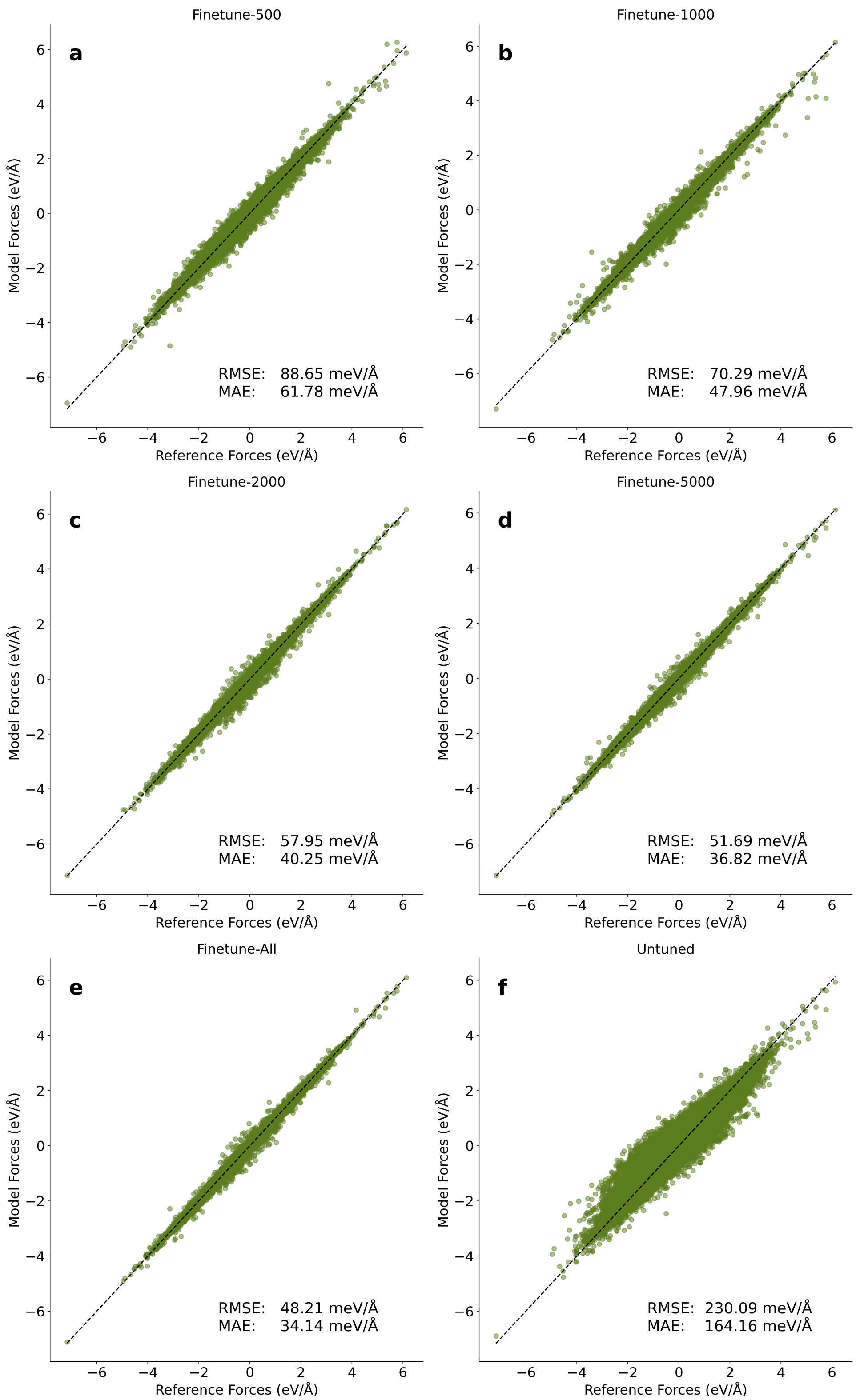

FIG. S11. Force parity plots for titanium atoms on the DFT hold-out test set. Panels (a)–(e) show finetuned models with increasing training set sizes, and panel (f) shows the untuned foundation model.

B. Classical Interfacial Water Structure

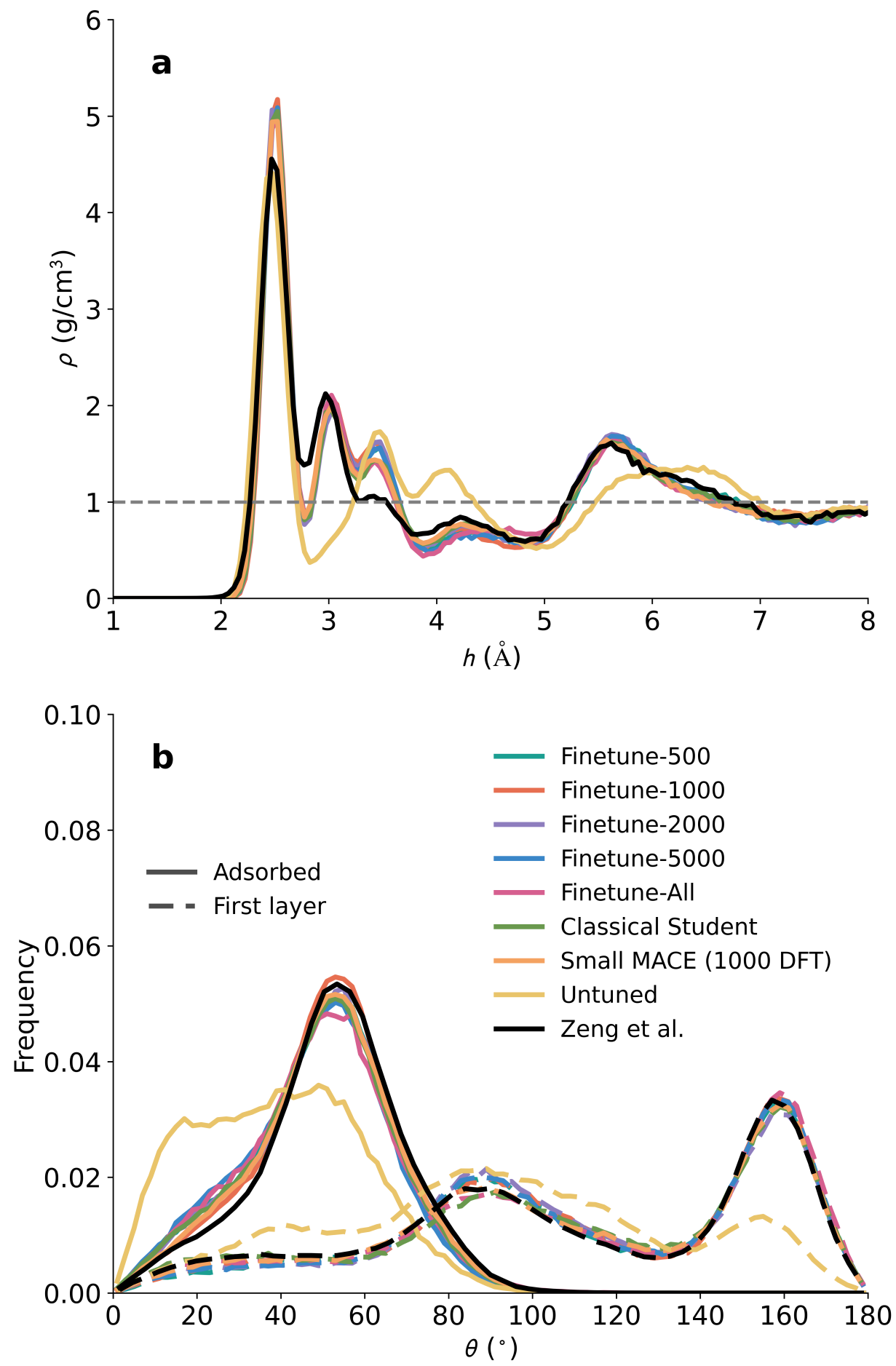

FIG. S12. Classical interfacial water structure from unbiased MD simulations at 330 K. (a) Water density profile as a function of height h above the $TiO_2(101)$ surface. The dashed horizontal line indicates the bulk water density. (b) Orientational distribution of the water dipole angle θ for adsorbed (solid lines) and first solvation layer (dashed lines) water molecules. Results are shown for all finetuned teacher models (Finetune-500 through Finetune-all), the classical student, the small MACE trained on 1000 DFT configurations, the untuned MACE-MP-0 foundation model, and the reference model of Zeng *et al.*[20]

C. Foundation-backbone comparison for TiO_2/water teacher construction

For the free-energy profiles in this comparison, each production trajectory was divided into three contiguous segments and a free energy profile was calculated independently from each segment. The solid curves in Figure S13 show the pointwise mean of the three segment profiles, and the shaded regions show their population standard deviation. These uncertainty bands quantify trajectory-segment variation for one finetuning realization at each data size and do not include training-seed uncertainty.

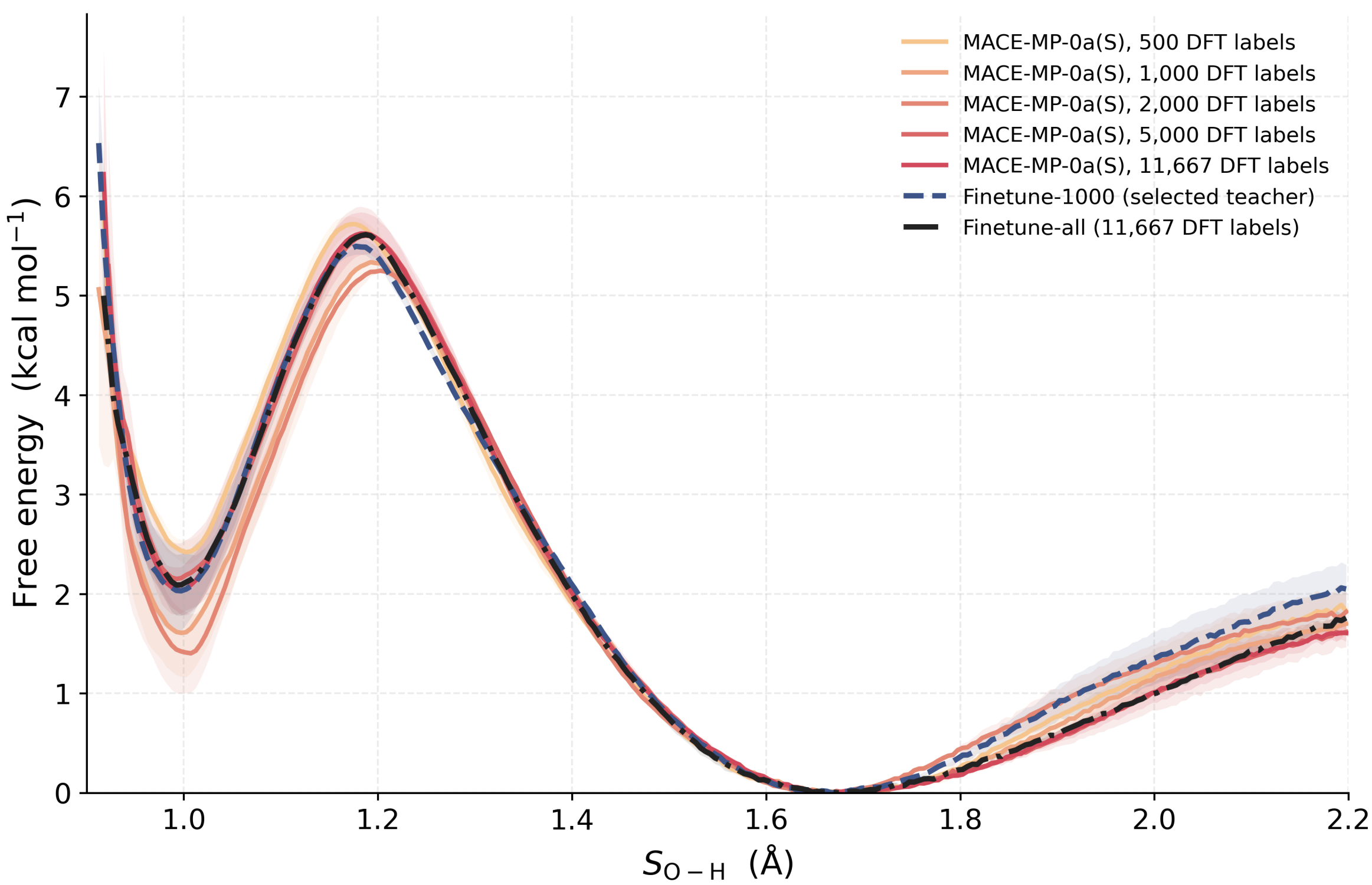

FIG. S13. Classical water-dissociation free energy profiles at the TiO_2(101)/water interface predicted by MACE-MP-0a(S) finetuned on 500, 1000, 2000, 5000, and 11,667 DFT-labeled configurations. Finetune-1000 and Finetune-all from the large-foundation comparison are shown as the selected teacher and high-data reference, respectively. Solid curves show the mean of three contiguous trajectory segments, and shaded regions show the corresponding population standard deviation.

TABLE S10. Dissociated-minus-molecular free energy differences, ΔG, and barriers relative to the molecular state, $G^{\ddagger}$, obtained with the large and small MACE-MP-0a foundation backbones at matched finetuning-set sizes. Values are the mean ± population standard deviation from three trajectory segments and are given in kcal mol^{-1}. The reference values of Zeng *et al.*[20] are included for comparison.

	ΔG		$G^{\ddagger}$	
DFT labels/reference	MACE-MP-0a(L)	MACE-MP-0a(S)	MACE-MP-0a(L)	MACE-MP-0a(S)
500	2.1 ± 0.3	2.42 ± 0.12	5.7 ± 0.2	5.72 ± 0.08
1,000	2.0 ± 0.4	1.61 ± 0.44	5.5 ± 0.1	5.34 ± 0.14
2,000	2.0 ± 0.1	1.40 ± 0.40	5.7 ± 0.1	5.25 ± 0.02
5,000	1.9 ± 0.2	2.15 ± 0.36	5.6 ± 0.2	5.60 ± 0.27
11,667	2.1 ± 0.3	2.03 ± 0.24	5.6 ± 0.1	5.62 ± 0.21
Zeng *et al.*	1.8 ± 0.3		5.4 ± 0.4	

D. Estimated temperature correction to the PIMD interfacial water-dissociation free energy

Following the notation used throughout the TiO_2/water discussion and in the reference study, we denote the free energy profile, the dissociated-minus-molecular free energy difference, and the free energy barrier by G, ΔG, and $G^{\ddagger}$, respectively. Because the present simulations were performed in the NVT ensemble, the computed profile is formally a Helmholtz free energy profile, A. At a common pressure, $\Delta G = \Delta A + p\Delta V_{\mathrm{rxn}}$. Given the weak compressibility of the condensed liquid and solid phases and the local nature of the proton-transfer event, we assume that the reaction-volume contribution is negligible and hence that $\Delta G \approx \Delta A$. We therefore retain the established G notation for the reported profiles while evaluating the temperature correction using the fixed-volume relation in Eq. (S4):

$$\Delta A = \Delta U - T\Delta S, \tag{S4}$$

where ΔA, ΔU, and ΔS denote dissociated-minus-molecular differences in Helmholtz free energy, internal energy, and entropy, respectively. We obtained ΔU_{330} from the PIMD internal energy estimator, $U = V + K_{\mathrm{cv}}$, where V is the potential energy and K_{cv} is the centroid-virial kinetic energy estimator.

The complete PIMD umbrella sampling trajectories were divided into the same three blocks used to estimate the free energy profile uncertainty. For each block i, the entropy difference was obtained from Eq. (S5):

$$\Delta S_{330,i} = \frac{\Delta U_{330,i} - \Delta A_{330,i}}{330\ \mathrm{K}}. \tag{S5}$$

Assuming that this entropy difference remains constant over 298–330 K gives the blockwise first-order estimate in Eq. (S6):

$$\Delta A_{298,i} \approx \Delta A_{330,i} + (330\ \mathrm{K} - 298\ \mathrm{K})\Delta S_{330,i}. \tag{S6}$$

Applying the temperature correction within each block retains the correlation between ΔA_{330} and ΔU_{330}. The three corrected block values, $\Delta A_{298,i}$, are 2.016, 1.491, and 1.541 kcal mol^{-1}.

The first-order extrapolation assumes that the entropy difference is temperature-independent over 298–330 K. Equivalently, it neglects the corresponding heat-capacity difference and treats the dissociated-minus-molecular internal energy difference as temperature-independent over this interval. The comparison additionally assumes that the reaction-volume contribution is negligible, such that $\Delta G \approx \Delta A$. These assumptions therefore make the estimate approximate and it cannot replace a direct PIMD umbrella sampling simulation at 298 K.

REFERENCES

[1] A. K. Soper, "The Radial Distribution Functions of Water as Derived from Radiation Total Scattering Experiments: Is There Anything We Can Say for Sure?" International Scholarly Research Notices **2013**, 279463 (2013).

[2] A. K. Soper, "The radial distribution functions of water and ice from 220 to 673 K and at pressures up to 400 MPa," Chemical Physics **258**, 121–137 (2000).

[3] N. O'Neill, B. X. Shi, W. J. Baldwin, W. C. Witt, G. Csányi, J. D. Gale, A. Michaelides, and C. Schran, "Towards Routine Condensed Phase Simulations with Delta-Learned Coupled Cluster Accuracy: Application to Liquid Water," Journal of Chemical Theory and Computation **21**, 11710–11720 (2025).

[4] V. Holten, C. E. Bertrand, M. A. Anisimov, and J. V. Sengers, "Thermodynamics of supercooled water," The Journal of Chemical Physics **136**, 094507 (2012).

[5] D. E. Hare and C. M. Sorensen, "The density of supercooled water. II. Bulk samples cooled to the homogeneous nucleation limit," The Journal of Chemical Physics **87**, 4840–4845 (1987).

[6] D. R. Lide, *CRC Handbook of Chemistry and Physics, 85th Edition* (CRC Press, 2004).

[7] D. E. Hare and C. M. Sorensen, "The density of supercooled water. ii. bulk samples cooled to the homogeneous nucleation limit," The Journal of Chemical Physics **87**, 4840–4845 (1987).

[8] G. S. Kell and E. Whalley, "The pvt properties of water. i. liquid water in the temperature range 0 to 150°c and at pressures up to 1 kb," Philosophical Transactions of the Royal Society of London. Series A **258**, 565–614 (1965).

[9] W. Gildseth, A. Habenschuss, and F. H. Spedding, "Precision measurements of densities

and thermal dilation of water between 5° and 80°c," Journal of Chemical & Engineering Data **17**, 402–409 (1972).

[10] A. Blahut, J. Hykl, P. Peukert, V. Vinš, and J. Hrubý, "Relative density and isobaric expansivity of cold and supercooled heavy water from 254 to 298 k and up to 100 mpa," The Journal of Chemical Physics **151**, 034505 (2019).

[11] F. J. Millero, R. Dexter, and E. Hoff, "Density and viscosity of deuterium oxide solutions from 5–70°c," Journal of Chemical & Engineering Data **16**, 85–87 (1971).

[12] T. L. Chang and L. H. Tung, "Density of heavy water," Nature **163**, 737 (1949).

[13] I.-C. Yeh and G. Hummer, "System-Size Dependence of Diffusion Coefficients and Viscosities from Molecular Dynamics Simulations with Periodic Boundary Conditions," The Journal of Physical Chemistry B **108**, 15873–15879 (2004).

[14] M. L. Huber, R. A. Perkins, A. Laesecke, D. G. Friend, J. V. Sengers, M. J. Assael, I. N. Metaxa, E. Vogel, R. Mareš, and K. Miyagawa, "New International Formulation for the Viscosity of H2O," Journal of Physical and Chemical Reference Data **38**, 101–125 (2009).

[15] A. Dehaoui, B. Issenmann, and F. Caupin, "Viscosity of deeply supercooled water and its coupling to molecular diffusion," Proceedings of the National Academy of Sciences **112**, 12020–12025 (2015).

[16] M. Ceriotti and D. E. Manolopoulos, "Efficient first-principles calculation of the quantum kinetic energy and momentum distribution of nuclei," Physical Review Letters **109**, 100604 (2012).

[17] B. Cheng, J. Behler, and M. Ceriotti, "Nuclear quantum effects in water at the triple point: Using theory as a link between experiments," The Journal of Physical Chemistry Letters **7**, 2210–2215 (2016).

[18] I. Batatia, P. Benner, Y. Chiang, A. M. Elena, D. P. Kovács, J. Riebesell, X. R. Advincula, M. Asta, M. Avaylon, W. J. Baldwin, F. Berger, N. Bernstein, A. Bhowmik, F. Bigi, S. M. Blau, V. Cărare, M. Ceriotti, S. Chong, J. P. Darby, S. De, F. Della Pia, V. L. Deringer, R. Elijošius, Z. El-Machachi, E. Fako, F. Falcioni, A. C. Ferrari, J. L. A. Gardner, M. J. Gawkowski, A. Genreith-Schriever, J. George, R. E. A. Goodall, J. Grandel, C. P. Grey, P. Grigorev, S. Han, W. Handley, H. H. Heenen, K. Hermansson, C. H. Ho, S. Hofmann, C. Holm, J. Jaafar, K. S. Jakob, H. Jung, V. Kapil, A. D. Kaplan, N. Karimitari, J. R. Kermode, P. Kourtis, N. Kroupa, J. Kullgren, M. C. Kuner, D. Kuryla, G. Liepuoniute, C. Lin, J. T. Margraf, I.-B. Magdău, A. Michaelides, J. H. Moore, A. A. Naik, S. P.

Niblett, S. W. Norwood, N. O'Neill, C. Ortner, K. A. Persson, K. Reuter, A. S. Rosen, L. A. M. Rosset, L. L. Schaaf, C. Schran, B. X. Shi, E. Sivonxay, T. K. Stenczel, C. Sutton, V. Svahn, T. D. Swinburne, J. Tilly, C. van der Oord, S. Vargas, E. Varga-Umbrich, T. Vegge, M. Vondrák, Y. Wang, W. C. Witt, T. Wolf, F. Zills, and G. Csányi, "A foundation model for atomistic materials chemistry," The Journal of Chemical Physics **163**, 184110 (2025).

[19] J. Klimeš, D. R. Bowler, and A. Michaelides, "Chemical accuracy for the van der Waals density functional," Journal of Physics: Condensed Matter **22**, 022201 (2009).

[20] Z. Zeng, F. Wodaczek, K. Liu, F. Stein, J. Hutter, J. Chen, and B. Cheng, "Mechanistic insight on water dissociation on pristine low-index TiO2 surfaces from machine learning molecular dynamics simulations," Nature Communications **14**, 6131 (2023).
